# Sex/gender differences in the association between behavioural factors and cancers: an umbrella review of systematic reviews with quantitative synthesis

**DOI:** 10.1186/s13293-025-00793-6

**Published:** 2025-11-23

**Authors:** Shelby Marozoff, Yannan Li, Nadia Mithani, Gabriela Kuczynski, Mohammad Ehsanul Karim, Arminee Kazanjian, Trevor J. B. Dummer

**Affiliations:** 1 School of Population and Public Health, University of British Columbia, 2206 East Mall, Vancouver, BC V6T 1Z3 Canada; 2 Centre for Advancing Health Outcomes, Vancouver, BC Canada

**Keywords:** Prevention, Sex, Gender, Cancer, Umbrella review, Diet, Physical activity

## Abstract

Many systematic reviews have summarized evidence on the association between behavioural factors and incident cancers. To date, there has been little synthesis of heterogeneity by sex/gender of this evidence.

An umbrella review was conducted of systematic reviews with quantitative synthesis (meta-analysis, meta-regression) examining the exposures of body size; physical activity; wholegrains, vegetables, fruit and beans; “fast foods”; red and processed meat; sugar sweetened drinks; dietary supplements; alcohol; tobacco; and sun exposure with incident non-sex-specific cancers. A search of Ovid MEDLINE, Ovid Embase, and Cochrane library from database inception to May 2023 was conducted. We calculated the proportion of systematic reviews that provided quantitative sex/gender findings (e.g., subgroup analyses) and summarized findings narratively. Methodological quality was appraised with the AMSTAR-2 tool.

From 13,227 records, 705 full-text systematic reviews were identified as meeting inclusion criteria. Of these, 361 (51.2%) reported quantitative sex/gender findings. The terms “sex” and “gender” were used interchangeably by 36.3% of the 361 systematic reviews and none reported findings for transgender, gender-diverse, or non-binary individuals. Overall, 98.6% (356/361) of systematic reviews were rated “critically low” with the AMSTAR-2 tool. Most of the 361 systematic reviews with quantitative sex/gender findings reported no statistically significant differences by sex/gender.

This umbrella review found conflation of sex and gender in systematic reviews of behavioural factors and non-sex-specific cancers and a lack of research among non-cisgender individuals. The existing evidence base is of critically low quality and our findings of no sex/gender-specific trends must be interpreted with caution.

**Supplementary Information:**

The online version contains supplementary material available at 10.1186/s13293-025-00793-6.

## Highlights

We examined sex/gender in the association of behavioural factors and cancer.

Around one-half of the included systematic reviews reported quantitative sex/gender results.

Around one-third of the included systematic reviews used terms “sex” and “gender” interchangeably.

Few statistically significant sex/gender differences in behavioural factors and cancer were found.

There is a lack of research on transgender, gender-diverse, and non-binary individuals.

**Supplementary Information:**

The online version contains supplementary material available at 10.1186/s13293-025-00793-6.

## Introduction

Globally, 40–50% of cancer cases could be prevented, through reductions in tobacco use, alcohol consumption, and overweight and obesity, among a range of other modifiable risk factors [1]. The World Cancer Research Fund/American Institute for Cancer Research (WCRF/AICR) published their Third Expert Report on Cancer Prevention Recommendations in 2020, specifying ten behavioural factors that impact cancer risk: maintaining a healthy body weight, physical activity, diets rich in whole grains, vegetables, fruit, and beans, diets low in “fast food”, red and processed meat, and sugar-sweetened drinks, limited alcohol consumption, non-use of dietary supplements, breastfeeding, as well as following the recommendations after a cancer diagnosis [2]. The WCRF/AICR also highlights smoking and sun exposure as important risk factors. These behavioural risk factors often demonstrate patterning by sex and gender. For instance, men have been found to have greater rates of tobacco use and a greater tobacco-related cancer incidence rate, relative to women [3]. Similarly, women tend to consume higher-quality diets than men [4]; the estimated percent of incident cancer cases attributable to low vegetable, low fruit, red meat, and processed meat consumption is 0.3%, 0.9%, 0.8%, and 0.7% in men, respectively and 0.2%, 0.6%, 0.5%, 0.4% in women, respectively [5].

Examining both sex and gender is important for understanding cancer etiology. Sex is considered a biological concept, primarily associated with anatomical and physiological features, such as chromosomes, hormones, and reproductive anatomy [6, 7]. Gender relates to the socially constructed roles, behaviours, expressions, and identities of women, men, girls, boys, and gender-diverse individuals [6]. Gender is a multidimensional concept including gender identity (how a person sees themselves), gender relations (how a person interacts with and is treated by others based on their attributed gender), gender expression (how a person presents their gender in society), behavioural norms, as well as the distribution of power and resources between genders in society, among other dimensions [7]. Sex and gender interact across the lifespan and influence cancer in numerous ways, such as through preventive behaviours and exposure to risk factors, sex hormones, immune responses, epigenetics, and others [8]. Additionally, sex and gender are often conflated in health research [6]. Per Springer et al., we use the term “sex/gender” to acknowledge that the biological dimensions of sex and the social dimensions of gender are entangled [9].

Many studies have reported differences in cancer incidence rates between individuals assigned male and female at birth for non-sex-specific cancers [10–12]. These non-sex-specific cancers are cancers that can develop at shared anatomic sites between sexes, such as the lungs and esophagus, but exclude sex-specific sites such as the testicles and ovaries. Males typically demonstrate greater incidence for the majority of cancer sites [13] and this trend is present across numerous geographical areas and persists across many age groups and racial and ethnic groups [14–16]. For instance, a recent analysis in the United States reported that males had an overall greater cancer incidence at almost all sites and age groups, with a notable exception of endocrine system cancers which have a greater incidence among females, particularly for the 20-29- and 30-39-year age groups [17]. Studies have also explored potential reasons for sex/gender differences in cancer incidence, finding that carcinogenic exposures and risk behaviours explain a portion of the difference [18, 19].

While many systematic reviews have examined the role of behavioural factors on incident cancers, an unknown proportion of them have quantitatively examined heterogeneity by sex/gender, for instance through subgroup analyses or meta-regression. The field of behavioural factors and cancer primary prevention is immense, making an umbrella review particularly relevant for summarizing data from systematic reviews, in order to provide a user-friendly summary for decision-makers [20]. To the best of our knowledge, no umbrella reviews have been conducted on this topic. Therefore, the aims of this umbrella review were two-fold: (1) to identify the proportion of systematic reviews with quantitative synthesis examining the association between behavioural factors and non-sex-specific cancer that report quantitative sex/gender findings and (2) to summarize how these associations between behavioural factors and non-sex-specific cancers vary according to sex/gender.

## Methods

This protocol of this review was registered in PROSPERO (CRD42023434890). Reporting was prepared in accordance with the PRIOR (Preferred Reporting Items for Overviews of Reviews) checklist [21](Supplemental Table 1).

### Search strategy

We searched Ovid MEDLINE, Ovid Embase, and the Cochrane library from database inception to May 2023 to identify systematic reviews with a quantitative synthesis (e.g., meta-analysis or meta-regression) of observational and interventional studies. Given the long latency period of cancer, as well as the unfeasibility of randomizing many behavioural exposures, we anticipated that most systematic reviews would be of observational studies; however we included systematic reviews of interventional studies given the strength of their evidence. The search strategy for Ovid MEDLINE is in Supplemental Tables 2 and was developed with consultation from a medical librarian. Searches in other databases were adapted from the original MEDLINE search strategy. The search was limited to full-text studies in English given the feasibility of reviewing the large volume of literature; there was no restriction regarding publication date.

### Inclusion criteria

We included all systematic reviews with a quantitative synthesis that evaluated the association between behavioural factors and incident non-sex-specific cancer, informed by the WCRF/AICR Cancer Prevention Recommendations. Specifically, the risk factors we included were body size; physical activity; wholegrains, vegetables, fruit and beans; “fast foods”; red and processed meat; sugar sweetened drinks; dietary supplements; alcohol; tobacco; and sun exposure. We did not include risk factors that are targeted towards specific populations, including mothers of infants (i.e. breastfeeding) and cancer survivors (i.e., following the Cancer Prevention Recommendations during or after treatment). We excluded risk factors measured using biomarkers, which are subject to inter-person variability due to absorption and metabolism, may not accurately reflect habitual behaviours, and are measured heterogeneously across studies, making synthesis challenging. More details regarding exposures of interest are provided in Supplemental Table 3.

Systematic reviews reporting on incident non-sex-specific cancer at any site were considered for inclusion; i.e., all cancers except for those occurring in only one biological sex: ovarian, cervical, uterine/endometrial, vaginal, vulvar, fallopian tube, penile, and testicular cancer. Prostate cancer was not considered, as we are aware of little research on cancer in Skene’s gland, which has similar functions to the prostate and has been referred to as the female prostate. For breast cancer, we additionally assumed systematic reviews included primary studies with only cisgender women, unless we were able to determine that at least one primary study included cisgender men. We included systematic reviews that reported a combined incidence and mortality outcome, given that historically for certain cancers, diagnosis and mortality often occur in quick succession, limiting the ability to study incidence and survival separately [22].However in such cases, if analyses on combined incidence and mortality outcomes and incidence-only outcomes were presented in a systematic review, we preferentially examined the incidence-only findings. Systematic reviews examining only cancer mortality outcome(s) were excluded.

The population was defined as all human adults ( > = 16 years). We required that systematic reviews not restrict to a single sex or gender. Systematic reviews were not limited by country of included studies or setting. However, those focusing on a specific disease group (e.g., diabetes) or a high-risk group (e.g., Lynch syndrome) were excluded, given the unique risk profiles.

Systematic reviews had to meet the following specific definition to be included: (1) have a research question; (2) report a search strategy with the sources searched or else report that the search strategy was available upon request and provide said strategy when contacted (two attempts were made to contact); (3) defined inclusion and/or exclusion criteria; (4) report the total number of included studies for the association of interest; and (5) synthesize the relevant findings quantitatively using meta-analysis or meta-regression. Systematic reviews that included case series or expert opinion were excluded as these sources provide overall lower quality evidence. Other umbrella reviews were included when the statistical analysis was done at the level of included primary studies and not at the level of included meta-analyses. All other types of reviews – such as narrative or scoping reviews – were excluded, as they do not provide a quantitative synthesis and do not meet the methodological standards for this umbrella review.

When our search identified two or more systematic reviews by the same team on the same association of interest, we excluded the earlier one. Otherwise, overlap in terms of populations, exposures, comparators, and outcomes in systematic reviews was not grounds for exclusion.

### Study selection and data extraction

Following the search, studies were consolidated in Covidence and duplicates were removed. Two reviewers (SM and either NM or YL) independently screened titles and abstracts, and subsequently, full-text articles against eligibility criteria (described above). Discrepancies were resolved by discussion and consensus amongst the three reviewers.

Data extraction was conducted in two phases. First, for all systematic reviews that met the inclusion criteria listed above, the following data was extracted into a spreadsheet by a reviewer (SM) and verified by a second reviewer (either NM or YL): authors, journal, title, URL, year of publication, funders and/or conflicts of interest, relevant exposure(s), relevant outcome(s), any published corrections, and whether quantitative sex/gender results were included. For sex/gender results, we accepted results from subgroup analyses or meta-regression that reported findings from two or more sex/gender groups for a particular association of interest. We did not accept sex/gender findings that were: (1) for an association not of interest (e.g., mortality); (2) reported only one sex or gender; (3) only comparing one sex or gender to a combined sex or gender group (e.g., males vs. combined males and females); (4) sex- or gender-specific epidemiologic measures calculated from a non-sex-specific effect estimate (e.g., attributable fractions for males and females); (5) no statistics reported in the systematic review, even if authors stated findings were statistically significant or not.

For all systematic reviews that did include quantitative sex/gender results, a reviewer (SM) extracted additional information into a separate spreadsheet, which was verified by a second reviewer (either NM, YL, or GK): exposure of interest, exposure comparison categories (e.g., highest vs. lowest, dose-response), outcome of interest, whether authors used the term “sex”, “gender”, or “both interchangeably”, number of primary studies or estimates included, effect estimates including confidence intervals (CIs) from sex/gender subgroup analyses, and measures of heterogeneity. For systematic reviews that conducted meta-regression with sex or gender included, we extracted the reported statistical output. For included systematic reviews that did not report this information, we contacted authors twice via email.

### Assessment of methodological quality

Systematic reviews that were deemed eligible for inclusion and that included quantitative sex/gender results were assessed for their methodological quality by a reviewer (SM) and verified by a second reviewer (NM, YL, or GK). Disagreements were addressed by discussion and consensus. We used the Assessing the Methodological Quality of Systematic Reviews-2 (AMSTAR-2) guidelines and checklist. AMSTAR-2 includes assessments of study eligibility criteria, identification and selection of studies, data collection methods, study appraisal methods and findings, and synthesis methods. It consists of 16 items, which can be rated “yes”, “no”, or “partial yes” [23]. An overall assessment of quality is derived based on seven critical items.

### Data summary

A narrative synthesis of the included systematic reviews was carried out by behavioural factor and cancer site. We did not conduct statistical pooling of results given the overlapping of primary studies within systematic reviews.

## Results

### Search results

**Fig. 1:**
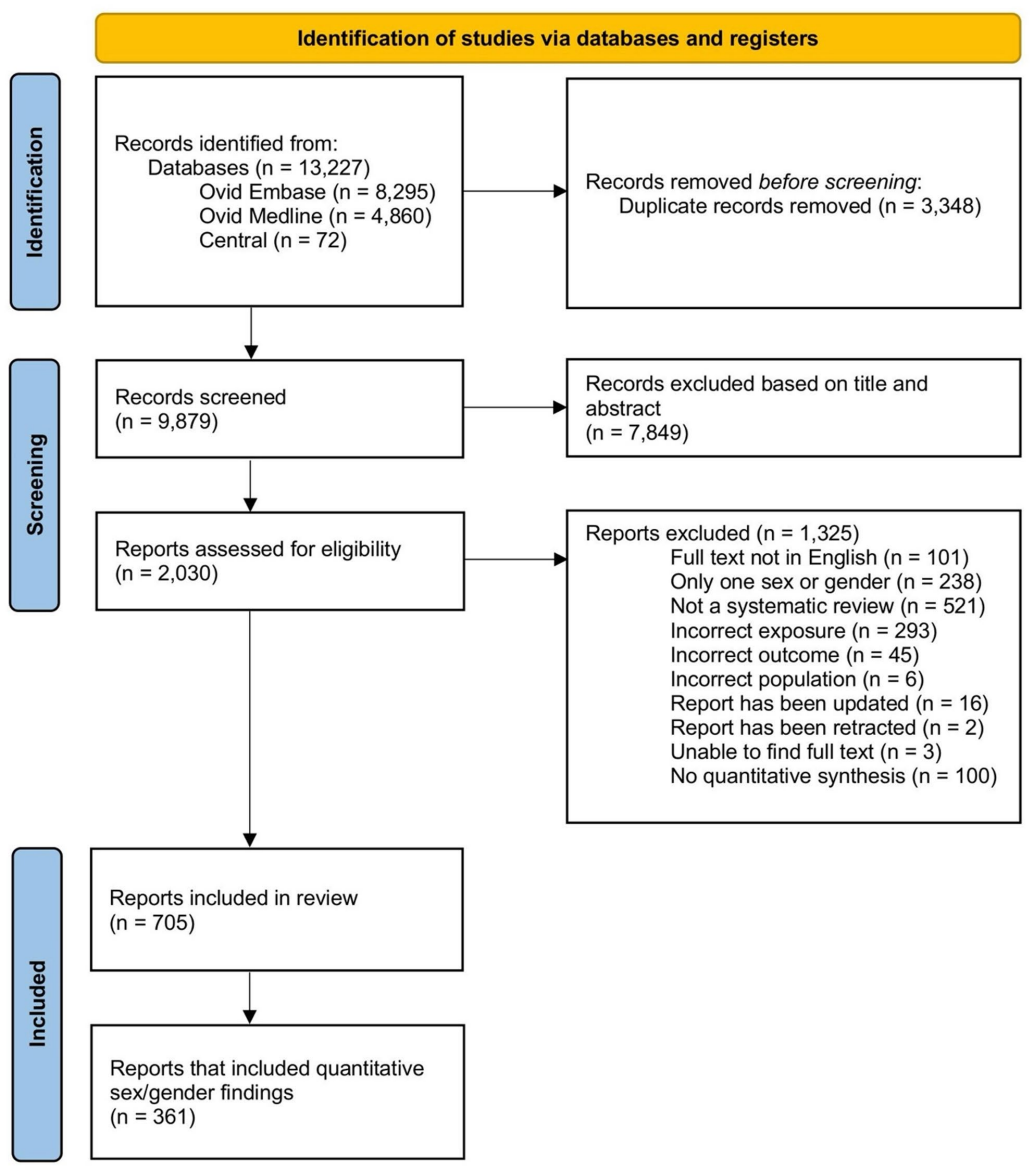
PRISMA flow diagram of the study selection process

A total of 13,227 records were identified from the three databases. Following exclusion of duplicates (*n* = 3,348), we identified 9,879 records. Following title and abstract screening, 7,849 records were excluded and 1,325 additional records were excluded after full-text screening. This resulted in a sample of 705 records (Fig. 1). The descriptive characteristics of the 705 systematic reviews are shown in Supplemental Table 4. A list of the excluded publications at the full-text screening stage and reasons for exclusion is available upon request from the corresponding author.

### Characteristics of 705 systematic reviews

**Fig. 2:**
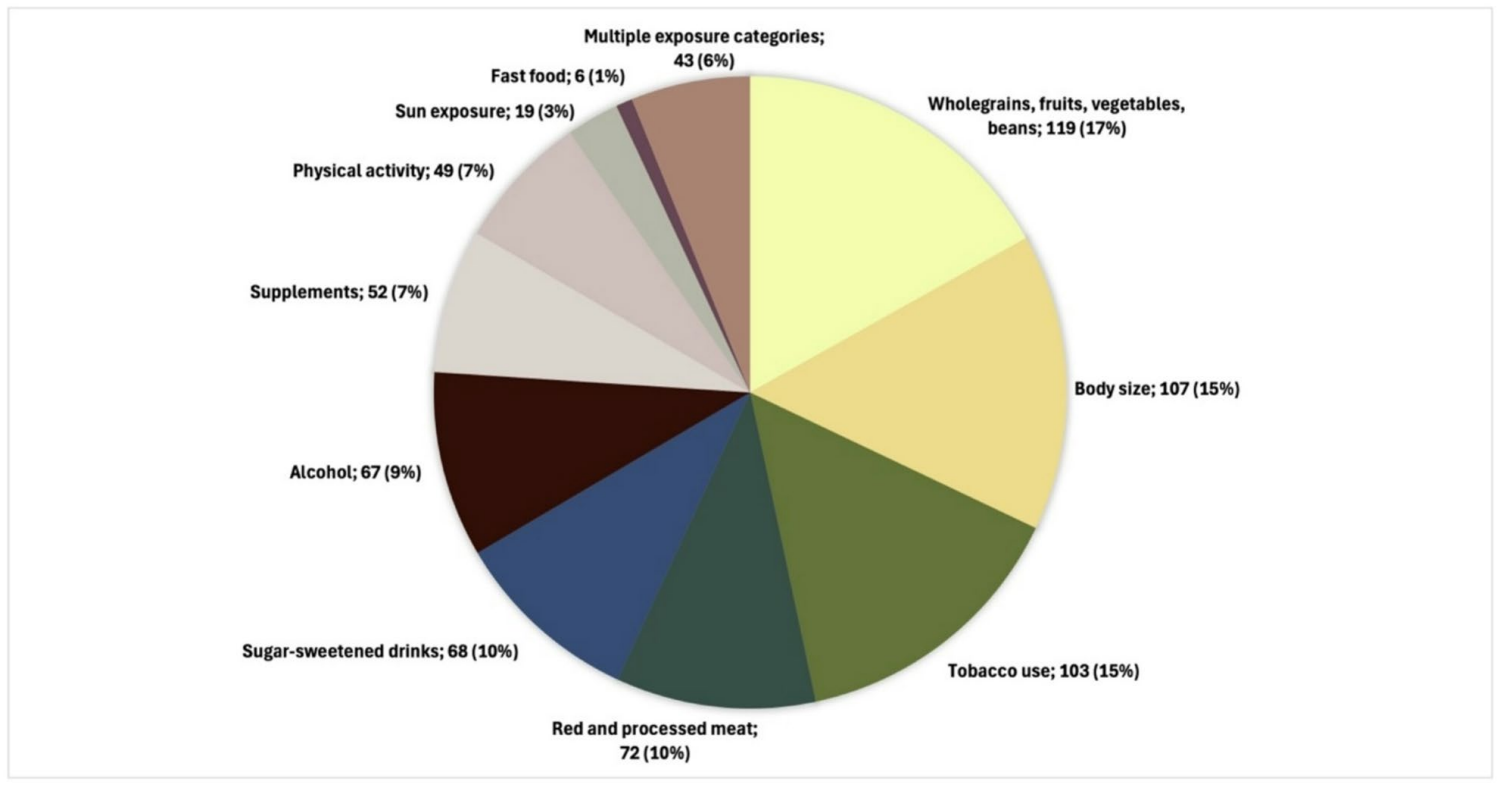
Graphical presentation of behavioural factors examined in 705 systematic reviews

Regarding exposure(s) of interest, the largest proportion of the included systematic reviews examined wholegrains, fruits, vegetables, or beans (*n* = 119; 16.9%), followed by body size (*n* = 107; 15.2%), and then tobacco use (*n* = 103; 14.6%). The smallest proportion of systematic reviews examined fast food (0.9%). Figure 2 presents the number of systematic reviews that examined each behavioural factor. Overall, 43 reviews examined a behavioural factor from more than one category. Reviews were published between 1997 and 2023 (Supplemental Fig. 1).

### Characteristics of 361 systematic reviews reporting sex/gender results

For the first aim of this umbrella review, we found that of the 705 systematic reviews, 361 (51.2%) reported quantitative sex/gender findings. We used a hierarchy for interpretation of subgroup analyses, with p-values from tests of interaction as the definitive measure, followed by relative risk ratios (RRRs) comparing two subgroups, then CI overlap for analyses with two subgroups. Subgroup analyses with three subgroups (e.g., males, females, and combined) without a p-value from a test of interaction could not be evaluated for statistical significance. For meta-regression, we also considered p-values as the definitive measure, followed by any other relevant measure, such as beta values with standard errors. Year of publication of reviews reporting sex/gender findings ranged 1997–2023. Descriptive characteristics of the 361 systematic reviews with quantitative sex/gender findings are in Supplemental Table 5.

Regarding sex and gender terminology, 158 reviews (43.8%) used the term “sex” throughout, 67 (18.6%) used the term “gender”, 131 (36.3%) used both “sex” and “gender” interchangeably, and 5 (1.4%) did not use either “sex” or “gender”. See Supplemental Fig. 2 for sex and gender terminology over time in the 361 systematic reviews reporting sex/gender results. No systematic reviews described the association between a behavioural factor and a cancer for transgender, gender-diverse, or non-binary individuals, referred to collectively as trans. We use the terms “men and women” or “males and females” hereafter, in accordance with what the authors of each systematic review use. The following sections address the second aim of this umbrella review, specifically focusing on the 361 systematic reviews reporting sex/gender results, and will be presented according to the WCRF/AICR recommendations.

### WCRF/AICR recommendation: be a healthy weight

**Fig. 3:**
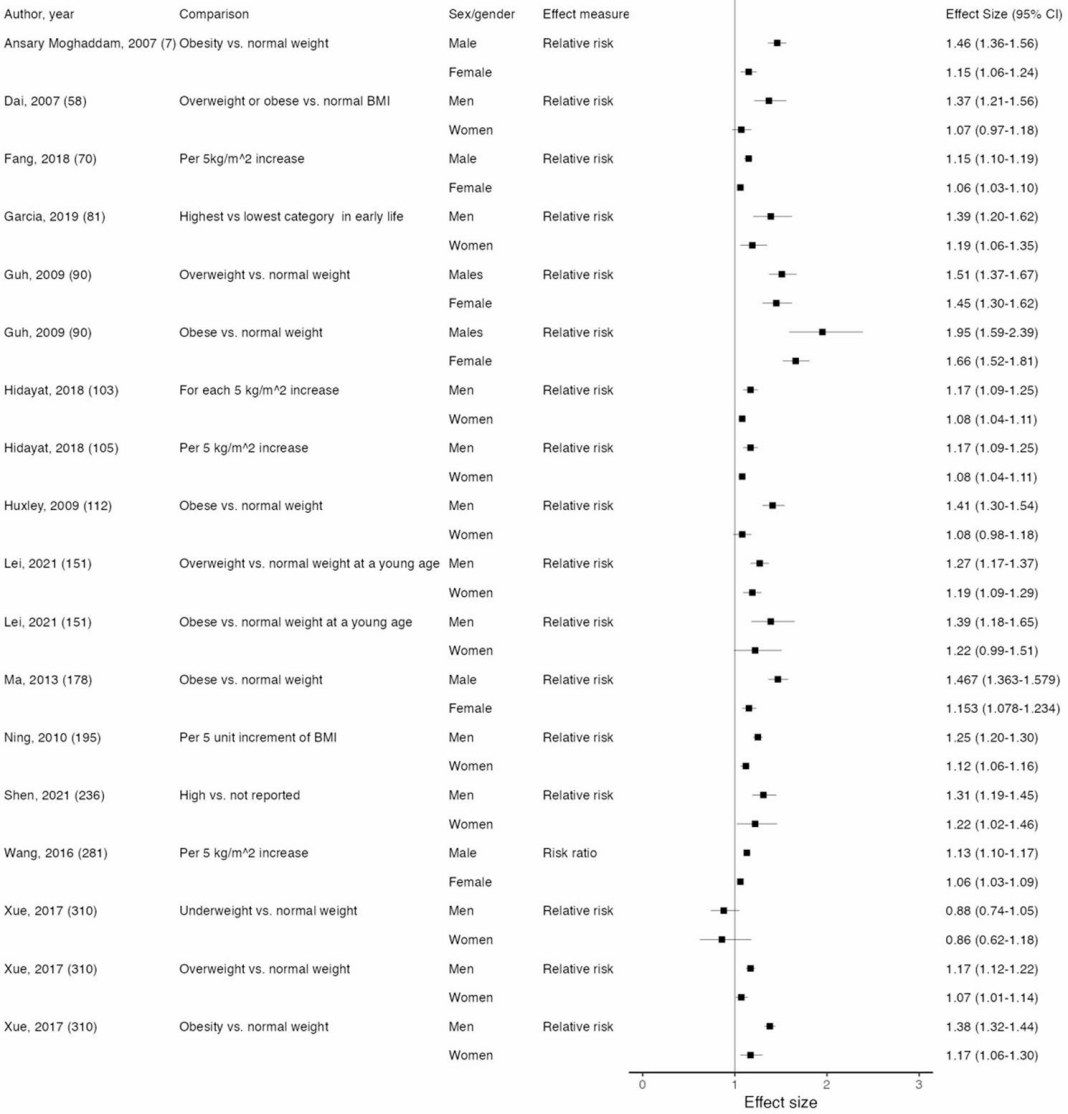
Forest plot of sex/gender subgroup analyses for body mass index and colorectal cancer. Reference for each subgroup analysis corresponds to those in Supplemental Table 5

We identified 72 systematic reviews with sex/gender findings that contributed results for body mass index (BMI). Table 1 lists the cancer sites that BMI was examined in association with, as well as the location of further information about each relevant systematic review in Supplemental Tables. No statistically significant sex/gender differences were identified for 21 of the included 35 cancer sites. Significant sex/gender differences for BMI were found for gastric cancer, gallbladder cancer, liver cancer, colorectal cancer (Fig. 3), colon cancer, rectal cancer, lung cancer, kidney cancer, brain cancer, melanoma, leukemia, Hodgkin’s lymphoma, acute myeloid leukemia, and chronic lymphocytic leukemia. For each association that demonstrated significant sex/gender differences, there were systematic reviews that also demonstrated no significant sex/gender differences.

**Table 1:** Summary of sex/gender findings from systematic reviews with quantitative synthesis for BMI and non-sex-specific cancers

Cancer	Corresponding systematic reviews in Supplemental Table 5	Narrative synthesis of sex/gender findings
Total cancer	70, 161, 186	No statistically significant findings
Esophageal cancer	70, 90, 310	No statistically significant findings
Esophageal squamous cell carcinoma	70, 214	No statistically significant findings
Esophageal adenocarcinoma	70, 103, 131, 214	No statistically significant findings
Esophageal and gastric cancer	131, 268, 281	No statistically significant findings
Gastric cancer	46, 70, 162, 214, 310, 317	Xue et al. reported that the RR associated with underweight was lower in women than men [24]. All other tests of interaction, subgroup analyses, RRRs, and meta-regression were not significant
Gallbladder cancer	70, 139, 157, 160, 164, 214, 255, 310	Three systematic reviews found an increased risk among women than for men for obesity from meta-regression [25], excess body weight from CI non-overlap [26], and underweight from an RRR [24], all relative to normal weight. Findings from five other systematic reviews found no sex/gender differences or were uninterpretable
Biliary tract cancer	200	No statistically significant findings
Cholangiocarcinoma	155, 157	No statistically significant findings
Liver cancer	45, 70, 103, 140, 214, 245, 281, 292, 310, 313, 314, 322	Sex/gender tests of interaction for subgroup analyses and RRRs from four systematic reviews indicated an increased risk for males/men relative to females/women for excess body weight, obesity, and underweight, all relative to normal weight or an unspecified comparison group [24, 27, 28, 29]. One systematic review reported a meta-regression wherein sex (female vs. mixed) explained some heterogeneity (p-value = 0.04) for the obesity exposure [30]. Findings from seven other systematic reviews found no sex/gender differences
Pancreatic cancer	24, 70, 90, 103, 135, 214, 281, 310	No statistically significant findings
Colorectal cancer	7, 58, 70, 81, 90, 103, 105, 112, 151, 178, 195, 236, 281, 310	The risk was higher for males/men than for females/women for obesity, overweight, and per 5-kg/m^2^ increase based on tests of interaction, RRRs, CI non-overlap, and meta-regression [24, 31–37]. Subgroup analyses and meta-regression from six other systematic reviews found no sex/gender differences (Fig. 3)
Colon cancer	7, 58, 70, 98, 137, 178, 195, 214, 310	Five systematic reviews reported an increased risk among males/men for obesity and per 5-kg/m^2^ increase, as compared with females/women [24, 34, 38–40], but no difference between sex/gender groups in four other systematic reviews
Proximal colon cancer	137, 217	No statistically significant findings
Distal colon cancer	137, 217	No statistically significant findings
Rectal cancer	7, 58, 70, 98, 137, 178, 195, 214, 217, 310	The risk was higher among men/males than women/females per 5-kg/m^2^ increase, as well as for overweight and for obesity vs. normal weight based on tests of interaction, CI non-overlap, RRRs, and meta-regression [24, 32, 38–40]. Five other systematic reviews reported no significant sex/gender differences
Lung cancer	67, 70, 214, 281, 310, 320, 346, 359	One of eight systematic reviews reported that the RR associated with overweight was higher in women than men (RRR: 1.14 (1.06–1.22)) [24]
Kidney cancer	23, 70, 90, 103, 169, 214, 279, 281, 310	Three systematic reviews reported increased risks among females/women relative to males/men for overweight, obesity, and per 5-kg/m^2^ increase, from RRRs, CI non-overlap, and meta-regression [24, 32, 41] and six systematic reviews reported no difference in sex/gender groups or were uninterpretable.
Bladder cancer	70, 211, 253, 271, 310	No statistically significant findings
Thyroid cancer	70, 103, 177, 214, 227, 281, 310, 326, 343, 348	No statistically significant findings
Brain cancer	70, 232, 338	One systematic review reported an increased risk per 5-kg/m^2^ increase among females compared to males, based on meta-regression and CI non-overlap [32] whereas the other two systematic reviews reported no sex/gender differences in subgroup analyses or were uninterpretable.
Glioma	232, 338	No statistically significant findings
Meningioma	194, 232, 233, 338	No statistically significant findings
Melanoma	70, 214, 230, 310	Subgroup analyses from two of four systematic reviews reported increased risks among males/men compared with females/women for overweight, obesity, combined overweight and obesity, and per 5-kg/m^2^ increase, all from CI non-overlap [40, 42]
Basal cell carcinoma	152	No statistically significant findings
Leukemia	1, 37, 70, 141, 209, 214, 310	Castillo et al. reported a 2.4% increase in incidence per 1-kg/m^2^ increase in men (*p* < 0.001), but no association in women in meta-regression analyses [43]. Larsson & Wolk reported an increased risk in men for obesity compared to normal weight, than in women from CI non-overlap in subgroup analysis [44]; the other five systematic reviews did not report significant sex/gender differences
Hodgkin’s lymphoma	1, 209	One of two systematic reviews reported non-overlap of CIs in a subgroup analysis per 5-kg/m^2^ increase, with the risk higher in women than in men [45], whereas the three subgroup analyses from the other review all demonstrated CI overlap
Non-Hodgkin’s lymphoma	1, 70, 104, 138, 142, 209, 214, 310	No statistically significant findings
Diffuse large beta cell lymphoma	1, 38, 103, 209	No statistically significant findings
Follicular lymphoma	1, 209	No statistically significant findings
Multiple myeloma	1, 70, 103, 136, 209, 214, 278, 310	No statistically significant findings
Acute myeloid leukemia	1, 37, 209	Castillo et al. reported a 3.8% increase (*p* < 0.001) per 1-kg/m^2^ increase in men but no linear relationship in women [43]. Subgroup analyses from two other systematic reviews each reported no significant sex/gender findings based on CI overlap
Chronic myeloid leukemia	1, 37	No statistically significant findings
Chronic lymphocytic leukemia	1, 37, 209	Castillo et al. reported a 1.3% increase (*p* = 0.02) per 1-kg/m^2^ increase in men but no linear relationship in women [43]. Subgroup analyses from two other systematic reviews each reported no significant sex/gender findings based on CI overlap
Acute lymphocytic leukemia	37	No statistically significant findings

BMI, body mass index; CI, confidence interval; RR, relative risk; RRR, relative risk ratio

Regarding exposures for body size other than BMI, we identified two systematic reviews for bariatric surgery, one for body surface area, four for weight, six for weight change, nine for waist circumference, one for hip circumference, and six for waist-to-hip ratio. A total of 24 of the 30 associations of interest between a body size exposure and a cancer site included no statistically significant sex/gender findings. The narrative syntheses for these associations of interest are provided in Table 2. Significant sex/gender findings were found for bariatric surgery and total cancer, weight change and colorectal cancer, weight change and colon cancer, weight change and thyroid cancer, waist circumference and colorectal cancer, and waist circumference and colon cancer. As with BMI, for most associations for which we identified significant sex/gender findings, we also identified systematic reviews with no significant sex/gender findings.

**Table 2:** Summary of sex/gender findings from systematic reviews with quantitative synthesis for body size exposures other than BMI and non-sex-specific cancers

Cancer	Corresponding systematic reviews in Supplemental Table 5	Narrative synthesis of sex/gender findings
*Bariatric surgery*		
Total cancer	259	One subgroup analysis found that males were at a greater risk than females (p for homogeneity = 0.021) [46]
Colorectal cancer	295	No statistically significant findings
*Body surface area*		
Melanoma	230	No statistically significant findings
*Weight*		
Thyroid cancer	227	No statistically significant findings
Basal cell carcinoma	152	No statistically significant findings
Non-Hodgkin’s lymphoma	1, 104	No statistically significant findings
Diffuse large beta cell lymphoma	1	No statistically significant findings
Follicular lymphoma	1	No statistically significant findings
*Weight change*		
Pancreatic cancer	122	No statistically significant findings
Colorectal cancer	44, 119, 224	CI non-overlap from subgroup analyses and meta-regression results from two systematic reviews report an increased risk among males/men relative to females/women for highest compared to lowest weight change, per 5-kg increment, high weight gain compared to stable weight, and per 1-kg increment [47, 48]. Meta-regression findings from one other systematic review indicated no significant difference between men and women
Colon cancer	44, 122, 224	One systematic review with two meta-regressions found an increased risk associated with high weight gain compared to stable weight and per 5-kg increase in body weight among men relative to women [48], whereas two other systematic reviews did not, based on tests of interaction and CI overlap
Rectal cancer	44, 224	No statistically significant findings
Kidney cancer	122, 145	No statistically significant findings
Thyroid cancer	122, 326	One of six subgroup analyses reported non-overlap of CIs with a higher risk in males than females for total weight gain < 10 kg (comparison group not reported) [49], with all other subgroup analyses and the meta-regression from the other systematic review reporting no significant sex/gender findings
*Waist circumference*		
Liver cancer	313	No statistically significant findings
Colorectal cancer	64, 90, 178	One of four subgroup analyses found an increased risk for obesity relative to normal weight among males compared to females, based on CI non-overlap [41], whereas the other subgroup analyses all demonstrated CI overlap
Colon cancer	58, 64, 137, 178	One of four subgroup analyses reported a significant difference with a higher risk in men than in women, per 10-cm increase (p for sex difference = 0.04) [39]
Rectal cancer	58, 64, 137, 178	No statistically significant findings
Bladder cancer	271	No statistically significant findings
Thyroid cancer	227	No statistically significant findings
Diffuse large beta cell lymphoma	1	No statistically significant findings
Follicular lymphoma	1	No statistically significant findings
*Hip circumference*		
Thyroid cancer	227	No statistically significant findings
*Waist-to-hip ratio*		
Liver cancer	313	No statistically significant findings
Colorectal cancer	64	No statistically significant findings
Colon cancer	58, 64, 137	No statistically significant findings
Rectal cancer	58, 64, 137	No statistically significant findings
Thyroid cancer	227	No statistically significant findings
Diffuse large beta cell lymphoma	1	No statistically significant findings
Follicular lymphoma	1	No statistically significant findings

BMI, body mass index; CI, confidence interval

### WCRF/AICR recommendation: be physically active

**Fig. 4:**
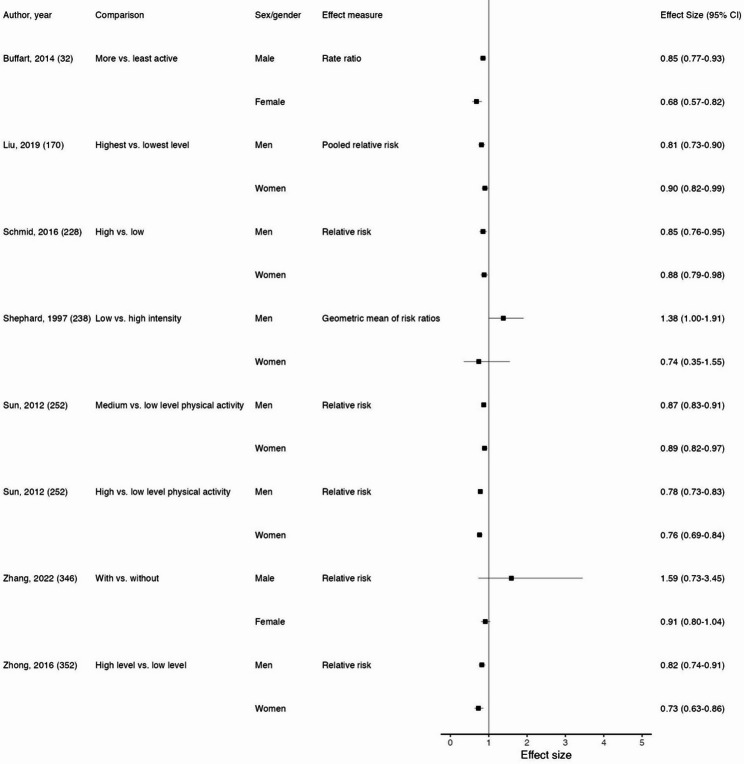
Forest plot of sex/gender subgroup analyses for overall physical activity and lung cancer. Reference for each subgroup analysis corresponds to those in Supplemental Table 5

This umbrella review identified 30 systematic reviews with sex/gender findings for overall physical activity and 27 different cancer sites. Findings are described in Table 3. Of the 27 associations of interest, there were no statistically significant findings regarding sex/gender differences for 22 of these associations. The associations for which we identified significant sex/gender differences in relation to overall physical activity were gastroesophageal cancer, gastric cancer, digestive system cancer, lung cancer (Fig. 4), and hematologic cancer.

Systematic reviews reporting on sex/gender differences for recreational physical activity in relation to incident cancer at seven sites were identified, based on 11 systematic reviews. None of these associations demonstrated statistically significant sex/gender results (Table 3). Similarly, we found no significant sex/gender findings for household physical activity and the one cancer outcome we identified (total cancer). We found systematic reviews examining sex/gender differences for sedentary behaviour in association with two cancer sites, of which none of the included systematic reviews reported statistically significant sex/gender findings.

**Table 3:** Summary of sex/gender findings from systematic reviews with quantitative synthesis for physical activity and non-sex-specific cancers

Cancer	Corresponding systematic reviews in Supplemental Table 5	Narrative synthesis of sex/gender findings
*Overall physical activity*		
Total cancer	238	No statistically significant findings
Esophageal cancer	19, 47	No statistically significant findings
Gastroesophageal cancer	19	One systematic review found an increased risk among men as compared with women in meta-regression (p-difference = 0.01) [50]
Gastric cancer	2, 19, 47, 207, 243	Of the five systematic reviews with sex/gender findings, only one found significant differences, with a higher RR in men than in women (p-difference = 0.02) [50]
Digestive system cancer	304	One systematic review conducted meta-regression, finding that the association of interest varied according to sex/gender (p-difference < 0.001), although subgroup analyses from the same systematic review demonstrated CI overlap [51]
Liver cancer	17	No statistically significant findings
Pancreatic cancer	15, 20	No statistically significant findings
Colorectal cancer	112, 235, 238	No statistically significant findings
Colon cancer	106, 238, 296	No statistically significant findings
Proximal colon cancer	28, 217	No statistically significant findings
Distal colon cancer	28, 217	No statistically significant findings
Rectal cancer	217, 221, 238	No statistically significant findings
Lung cancer	32, 170, 228, 238, 252, 346, 352	Of the seven systematic reviews that provided sex/gender findings, only Buffart et al. reported a significant difference between sex/gender groups (p-difference = 0.03) [52], suggesting a greater risk in males than females (Fig. 4)
Kidney cancer	18	No statistically significant findings
Bladder cancer	120	No statistically significant findings
Thyroid cancer	225	No statistically significant findings
Meningioma	194	No statistically significant findings
Glioma	194	No statistically significant findings
Melanoma	21	No statistically significant findings
Hematologic cancer	118	The one identified systematic review reported an inverse relation in women, but not in men (p-difference = 0.03) [53]
Leukemia	118	No statistically significant findings
Hodgkin’s lymphoma	60, 118	No statistically significant findings
Non-Hodgkin’s lymphoma	60, 118	No statistically significant findings
Diffuse large beta cell lymphoma	60, 118	No statistically significant findings
Follicular lymphoma	60, 118	No statistically significant findings
Multiple myeloma	118	No statistically significant findings
Chronic lymphocytic leukemia/small lymphocytic leukemia	60, 118	No statistically significant findings
*Recreational physical activity*		
Total cancer	82, 166, 238	No statistically significant findings
Gastric cancer	207	No statistically significant findings
Pancreatic cancer	15, 72	No statistically significant findings
Colorectal cancer	166, 179	No statistically significant findings
Colon cancer	82, 97, 179, 221, 238	No statistically significant findings
Rectal cancer	82, 97, 179	No statistically significant findings
Lung cancer	30, 258	No statistically significant findings
*Household physical activity*		
Total cancer	239	No statistically significant findings
*Sedentary behaviour*		
Colon cancer	102, 226	No statistically significant findings
Rectal cancer	102	No statistically significant findings

RR, relative risk

### WCRF/AICR recommendation: eat wholegrains, vegetables, fruit and beans

We identified two systematic reviews with sex/gender findings for the exposure of wholegrains, both of which were for the colorectal cancer outcome. For fibre, we found three systematic reviews with sex/gender findings, one each for pancreatic cancer, colon cancer, and rectal cancer. A total of six systematic reviews with sex/gender findings were identified for combined fruits and vegetables, 30 for vegetables only, and 18 for fruits only. One systematic review with sex/gender findings was identified for beans. All of the above systematic reviews for dietary exposures reported no statistically significant sex/gender differences, with the exception of one systematic review examining vegetables consumption and non-Hodgkin’s lymphoma. Findings for wholegrains, fibre, vegetables, fruit, and beans are described in Table 4.

We also included systematic reviews examining dietary indices and data-driven dietary patterns. Sex/gender findings were identified from one systematic review for any dietary index, four systematic reviews for the DASH (Dietary Approaches to Stop Hypertension) diet, four systematic reviews for the Dietary Inflammatory Index, one systematic review for the Healthy Eating Index, six systematic reviews for the Mediterranean diet, two systematic reviews for a Western/unhealthy dietary pattern, and three systematic reviews for a prudent/healthy dietary pattern (Table 5). In total, we examined 27 associations of interest for dietary indices or patterns and cancer sites, of which only one included a statistically significant sex/gender difference – that of the DASH diet and colorectal cancer.

**Table 4:** Summary of sex/gender findings from systematic reviews with quantitative synthesis for wholegrains, vegetables, fruit, beans and non-sex-specific cancers

Cancer	Corresponding systematic reviews in Supplemental Table 5	Narrative synthesis of sex/gender findings
*Wholegrains*		
Colorectal cancer	94, 229	No statistically significant findings
*Fibre*		
Pancreatic cancer	197	No statistically significant findings
Colon cancer	85	No statistically significant findings
Rectal cancer	86	No statistically significant findings
*Combined vegetables and fruit*		
Total cancer	10	No statistically significant findings
Pancreatic cancer	350	No statistically significant findings
Lung cancer	289, 318	No statistically significant findings
Bladder cancer	303, 321	No statistically significant findings
*Vegetables*		
Total cancer	10, 340	No statistically significant findings
Oral cancer	202	No statistically significant findings
Gastric cancer	192, 286, 298	No statistically significant findings
Liver cancer	92	No statistically significant findings
Pancreatic cancer	300, 350	No statistically significant findings
Colorectal cancer	49, 109, 216, 229, 264, 270, 299, 355	No statistically significant findings
Colon cancer	109, 357	No statistically significant findings
Rectal cancer	109, 357	No statistically significant findings
Lung cancer	216, 283, 289, 346	No statistically significant findings
Urothelial cancer	175	No statistically significant findings
Kidney cancer	341, 347	No statistically significant findings
Bladder cancer	303, 321, 327	No statistically significant findings
Thyroid cancer	171	No statistically significant findings
Non-Hodgkin’s lymphoma	43	One systematic review reported significant subgroup differences between studies of men, women, and combined men and women (p-difference = 0.02) [54]
Non-digestive tract cancer	91	No statistically significant findings
*Fruit*		
Total cancer	10	No statistically significant findings
Oral cancer	202	No statistically significant findings
Gastric cancer	192, 286	No statistically significant findings
Pancreatic cancer	301, 350	No statistically significant findings
Colorectal cancer	216, 229	No statistically significant findings
Lung cancer	68, 216, 280, 283, 289, 346	No statistically significant findings
Kidney cancer	237, 341	No statistically significant findings
Bladder cancer	303, 321	No statistically significant findings
Non-Hodgkin’s lymphoma	43	No statistically significant findings
*Beans*		
Colorectal cancer	229	No statistically significant findings

**Table 5:** Summary of sex/gender findings from systematic reviews with quantitative synthesis for dietary indices and dietary patterns and non-sex-specific cancers

Cancer	Corresponding systematic reviews in Supplemental Table 5	Narrative synthesis of sex/gender findings
*Any dietary index*		
Total cancer	188	No statistically significant findings
*DASH diet*		
Colorectal cancer	6, 185, 187, 257	One subgroup analysis by Moazzen et al. had non-overlapping CIs (odds ratio (OR) men: 0.76 (95% CI: 0.73–0.80); OR women: 0.85 (95% CI: 0.81–0.90) [55]
Colon cancer	6	No statistically significant findings
Rectal cancer	6	No statistically significant findings
*Dietary inflammatory index*		
Upper gastrointestinal cancer	184	No statistically significant findings
Pancreatic cancer	116	No statistically significant findings
Colorectal cancer	116, 185, 240	No statistically significant findings
Colon cancer	240	No statistically significant findings
Rectal cancer	240	No statistically significant findings
*Healthy eating index*		
Colorectal cancer	185	No statistically significant findings
*Mediterranean diet*		
Upper gastrointestinal cancer	184	No statistically significant findings
Pancreatic cancer	198	No statistically significant findings
Colorectal cancer	185, 189, 353	No statistically significant findings
Lung cancer	14	No statistically significant findings
*Western/unhealthy dietary pattern*		
Esophageal squamous cell carcinoma	89	No statistically significant findings
Gastric cancer	89	No statistically significant findings
Pancreatic cancer	89	No statistically significant findings
Colorectal cancer	83, 89	No statistically significant findings
Colon cancer	83, 89	No statistically significant findings
Rectal cancer	83, 89	No statistically significant findings
*Prudent/healthy dietary pattern*		
Esophageal squamous cell carcinoma	89	No statistically significant findings
Gastric cancer	89	No statistically significant findings
Pancreatic cancer	89	No statistically significant findings
Colorectal cancer	83, 89	No statistically significant findings
Colon cancer	83, 89	No statistically significant findings
Rectal cancer	83, 89	No statistically significant findings
Lung cancer	254	No statistically significant findings

CI, confidence interval; DASH, dietary approaches to stop hypertension; OR, odds ratio

### WCRF/AICR recommendation: limit ‘fast foods’

This umbrella review identified one systematic review with sex/gender differences for the exposure of glycemic index or load and one for the exposure of processed foods (Table 6). Neither reported statistically significant sex/gender differences in the associations of interest.

**Table 6:** Summary of sex/gender findings from systematic reviews with quantitative synthesis for fast food and dietary patterns and non-sex-specific cancers

Cancer	Corresponding systematic reviews in Supplemental Table 5	Narrative synthesis of sex/gender findings
*Glycemic index or load*		
Diabetes-related cancers	51	No statistically significant findings
*Processed foods*		
Nasopharyngeal cancer	74	No statistically significant findings

### WCRF/AICR recommendation: limit red and processed meat

For the exposure of meat, we identified one systematic review with sex/gender findings for all meat, two for red and processed meat combined, 16 for red meat, 15 for processed meat, five for white meat not including fish, one for white meat including fish, eight for fish, one for shellfish, and two for salted fish (Table 7). From these systematic reviews 33 associations of interest were reported. Only two of the associations of interest included statistically significant sex/gender findings: between fish and colorectal cancer and salted fish and gastric cancer. For both associations, we also identified systematic reviews that reported no significant sex/gender differences.

**Table 7:** Summary of sex/gender findings from systematic reviews with quantitative synthesis for meat and non-sex-specific cancers

Cancer	Corresponding systematic reviews in Supplemental Table 5	Narrative synthesis of sex/gender findings
*All meat*		
Renal cancer	71	No statistically significant findings
*Red and processed meat*		
Colorectal cancer	5, 39	No statistically significant findings
Colon cancer	39	No statistically significant findings
*Red meat*		
Esophageal cancer	52	No statistically significant findings
Gastric cancer	127, 250, 358	No statistically significant findings
Pancreatic cancer	128, 143, 349	No statistically significant findings
Colorectal cancer	5, 39, 134, 229	No statistically significant findings
Colon cancer	5, 39, 134	No statistically significant findings
Rectal cancer	5	No statistically significant findings
Lung cancer	87, 312, 319	No statistically significant findings
Bladder cancer	153	No statistically significant findings
Non-Hodgkin’s lymphoma	316	No statistically significant findings
*Processed meat*		
Esophageal cancer	52	No statistically significant findings
Gastric cancer	127, 297, 358	No statistically significant findings
Pancreatic cancer	128, 143, 349	No statistically significant findings
Colorectal cancer	4, 39, 96, 134, 229	No statistically significant findings
Colon cancer	4, 39, 96, 134	No statistically significant findings
Rectal cancer	4, 96, 134	No statistically significant findings
Lung cancer	312, 319	No statistically significant findings
Non-Hodgkin’s lymphoma	316	No statistically significant findings
*White meat*,* not including fish*		
Gastric cancer	127	No statistically significant findings
Pancreatic cancer	80, 128	No statistically significant findings
Colorectal cancer	34	No statistically significant findings
Lung cancer	319	No statistically significant findings
*White meat*,* including fish*		
Lung cancer	319	No statistically significant findings
*Fish*		
Gastrointestinal cancer	328	No statistically significant findings
Pancreatic cancer	80	No statistically significant findings
Colorectal cancer	33, 84, 229	One systematic review reported significant subgroup differences comparing women and men (*p* = 0.04) per daily 100-gram increase of fish [56], although three other subgroup analyses for this association from two systematic reviews had CI overlap or were uninterpretable
Colon cancer	33	No statistically significant findings
Rectal cancer	33	No statistically significant findings
Lung cancer	176, 249, 319	No statistically significant findings
*Shellfish*		
Thyroid cancer	171	No statistically significant findings
*Salted fish*		
Gastric cancer	297, 325	The systematic review by Wu et al. reported a statistically significant interaction test comparing men and women for moderate vs. low intake (*p* = 0.035) and a ratio between subgroups of 1.52 (95% CI: 1.03–2.25) [57], suggesting greater risk among men than women. The test for interaction and ratio between subgroups for high vs. low intake from the same systematic review and two meta-regressions from the other systematic review were not statistically significant

CI, confidence interval

### WCRF/AICR recommendation: limit sugar sweetened drinks

**Fig. 5:**
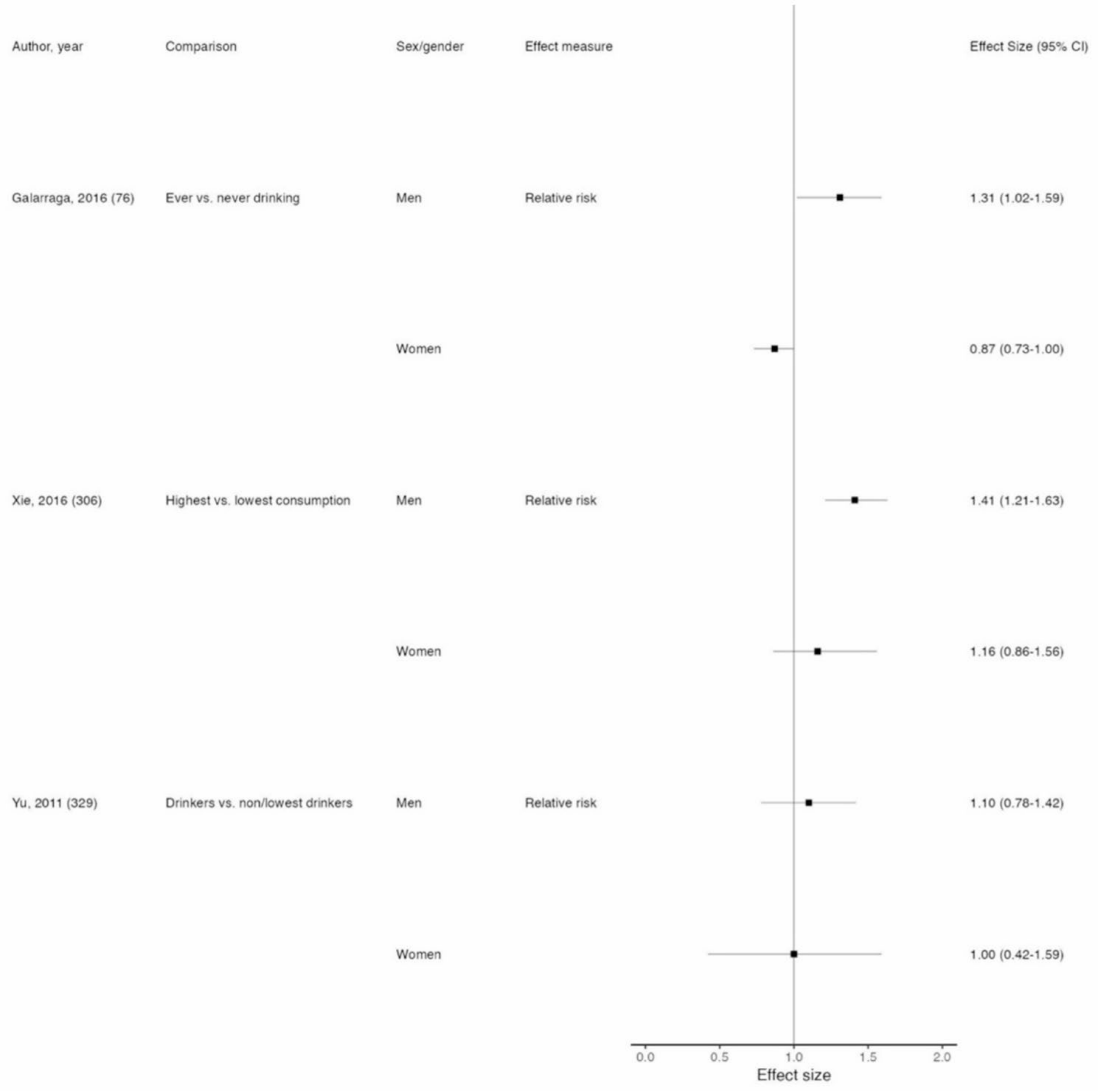
Forest plot of sex/gender subgroup analyses for coffee consumption and lung cancer. Reference for each subgroup analysis corresponds to those in Supplemental Table 5

We did not identify any systematic reviews that reported quantitative sex/gender findings for sugar sweetened drinks specifically. We did identify 27 systematic reviews with sex/gender findings for coffee consumption, wherein a significant sex/gender difference was found only for the association between coffee and lung cancer (Fig. 5). Table 8 reports the narrative synthesis for sex/gender findings in relation to coffee and tea consumption. For tea consumption, we identified 21 systematic reviews with sex/gender findings. No associations of interest for tea were statistically significant, except for one of two systematic reviews for the colorectal cancer outcome. In both cases, we also identified systematic reviews with no significant sex/gender findings.

**Table 8:** Summary of sex/gender findings from systematic reviews with quantitative synthesis for non-alcoholic beverages and non-sex-specific cancers

Cancer	Corresponding systematic reviews in Supplemental Table 5	Narrative synthesis of sex/gender findings
*Coffee*		
Total cancer	329	No statistically significant findings
Esophageal cancer	339	No statistically significant findings
Gastric cancer	61, 156, 163, 248, 306, 329, 336	No statistically significant findings
Liver cancer	29, 88, 222, 329	No statistically significant findings
Pancreatic cancer	63, 266, 329	No statistically significant findings
Colorectal cancer	3, 77, 117, 183, 329	No statistically significant findings
Colon cancer	3, 77, 117, 183	No statistically significant findings
Rectal cancer	3, 77, 117, 183	No statistically significant findings
Lung cancer	76, 306, 329	Galarraga & Boffetta found an RR among men of 1.31 (95% CI: 1.02–1.59) and among women of 0.87 (95% CI: 0.73-1.00) for those who consumed coffee compared with those who did not (Fig. 5) [58]. The other two subgroup analyses demonstrated CI overlap
Urinary tract cancer	334	No statistically significant findings
Kidney cancer	215, 329	No statistically significant findings
Bladder cancer	59, 108, 302, 329, 356	No statistically significant findings
Melanoma	165, 324	No statistically significant findings
Glioma	204	No statistically significant findings
*Tea*		
Oral cancer	167, 354	No statistically significant findings
Esophageal cancer	223, 351	No statistically significant findings
Biliary tract cancer	307	No statistically significant findings
Gastric cancer	345	No statistically significant findings
Liver cancer	110, 193, 242	No statistically significant findings
Pancreatic cancer	40, 293, 335, 345	No statistically significant findings
Colorectal cancer	251, 360	A test for interaction comparing men and women was significant, for highest vs. non/lowest black tea consumption (*p* = 0.03), indicating a protective effect in women not seen in men [59]. The subgroup analysis in this same systematic review for green tea was not significant, nor were subgroup analyses from the other systematic review
Colon cancer	345, 360	No statistically significant findings
Rectal cancer	345, 360	No statistically significant findings
Lung cancer	93, 256, 282	No statistically significant findings
Urinary tract cancer	334	No statistically significant findings
Bladder cancer	108, 287, 301, 345	No statistically significant findings

CI, confidence interval; RR, relative risk

### WCRF/AICR recommendation: do not use supplements for cancer prevention

Sex/gender findings were identified in one systematic review for selenium supplements, two systematic reviews for calcium supplements, three systematic reviews for folic acid supplements, two systematic reviews for beta-carotene supplements, one systematic review for omega-3 fatty acid supplements, one systematic review for vitamin B supplements, two systematic reviews for vitamin C supplements, two systematic reviews for vitamin D supplements, and one systematic review for multiple supplements (specifically, a combination of vitamins A, C, and E, with selenium, and zinc). Findings are described in Table 9. Systematic reviews were found identifying significant sex/gender differences for selenium supplements and total cancer, beta-carotene supplements and gastric cancer, as well as vitamin D supplements and colorectal cancer. For the other 19 associations of interest between one or more dietary supplements and cancer sites, no statistically significant sex/gender findings were identified.

**Table 9:** Summary of sex/gender findings from systematic reviews with quantitative synthesis for dietary supplements and non-sex-specific cancers

Cancer	Corresponding systematic reviews in Supplemental Table 5	Narrative synthesis of sex/gender findings
*Selenium supplements*		
Total cancer	16	The test for interaction for the identified systematic review suggests selenium supplementation had a beneficial effect in reducing cancer incidence in men (*p* < 0.001 for interaction by sex) [60]
*Calcium supplements*		
Total cancer	31	No statistically significant findings
Colorectal cancer	31, 121	No statistically significant findings
*Folic acid supplements*		
Total cancer	213, 276	No statistically significant findings
Colorectal cancer	212	No statistically significant findings
*Beta-carotene supplements*		
Total cancer	65	No statistically significant findings
Gastric cancer	65	We identified one subgroup analysis from one systematic review for beta-carotene supplements vs. placebo, which demonstrated a greater risk among studies with a majority of men (RR: 1.34 (95% CI: 1.06–1.70) than a majority of women (RR: 0.84 (95% CI: 0.71-1.00)) based on non-overlap of CIs [61]
Pancreatic cancer	65	No statistically significant findings
Colorectal cancer	65	No statistically significant findings
Lung cancer	65, 129	No statistically significant findings
Melanoma	65	No statistically significant findings
Non-melanoma skin cancer	65	No statistically significant findings
Basal cell skin cancer	65	No statistically significant findings
Squamous cell skin cancer	65	No statistically significant findings
*Omega-3 fatty acid supplements*		
Total cancer	344	No statistically significant findings
*Vitamin B supplements*		
Total cancer	342	No statistically significant findings
*Vitamin C supplements*		
Total cancer	56, 144	No statistically significant findings
Lung cancer	56	No statistically significant findings
*Vitamin D supplements*		
Colorectal cancer	27, 100	Of two subgroup analyses, one demonstrated non-overlap of CIs with a hazard ratio of 0.65 (0.50–0.85) among men and 1.05 (0.91–1.21) among women [62]. The other demonstrated overlap of sex/gender CIs
Colon cancer	27	No statistically significant findings
*Multiple supplements*		
Total cancer	56	No statistically significant findings
Lung cancer	56	No statistically significant findings

CI, confidence interval; RR, relative risk

### WCRF/AICR recommendation: limit alcohol consumption

We identified sex/gender findings from 45 systematic reviews assessing the association between alcohol consumption and 25 cancer sites (Table 10). Of these associations of interest between alcohol consumption and a cancer site, 19 were found to have no significant sex/gender differences. Systematic reviews with significant sex/gender differences were found for alcohol consumption and oral cavity and pharynx cancer, esophageal cancer, colorectal cancer, rectal cancer, bladder cancer, and brain cancer. Two systematic reviews were identified for alcohol cessation: one in reference to gastric cancer and one multiple myeloma. There were no significant sex/gender differences identified for alcohol cessation.

**Table 10:** Summary of sex/gender findings from systematic reviews with quantitative synthesis for alcohol and non-sex-specific cancers

Cancer	Corresponding systematic reviews in Supplemental Table 5	Narrative synthesis of sex/gender findings
*Alcohol consumption*		
Oral cavity and pharynx cancer	12, 13, 50, 55, 267	One subgroup analysis by Turati et al. reported significant differences between men and women (p for heterogeneity < 0.001) with a greater risk among men (63), whereas the eight other subgroup analyses demonstrated non-significant tests of interaction and/or overlap of CIs. Also, Corrao et al. reported a significant effect of gender in modifying the linear effect of alcohol, with women presenting higher slopes than men (beta = 0.01655; Z value = 5.375) [64]
Esophageal cancer	55, 330	The one subgroup analysis examining the highest vs. lowest alcohol intake found a higher RR for men than for women, with no overlap of CIs [65]
Esophageal squamous cell carcinoma	12, 13, 330	No statistically significant findings
Esophageal adenocarcinoma and gastric cardia cancer	13, 262	No statistically significant findings
Gastric cancer	13, 50, 62, 95, 99, 261, 284	No statistically significant findings
Liver cancer	12, 13, 53, 55, 269	No statistically significant findings
Pancreatic cancer	13, 50, 260, 290	No statistically significant findings
Colorectal cancer	12, 13, 50, 73, 190, 308, 337	Among the 15 subgroup analyses, Bagnardi et al. found that the association was stronger in men than in women (p for heterogeneity across light, moderate, and heavy alcohol intake = 0.010) [66]. Also, Fedirko et al. reported significant differences between males and females for alcohol drinkers vs. non/occasional drinkers (p from test of homogeneity = 0.001), as well as for moderate alcohol drinkers vs. non/occasional drinkers(p from test of homogeneity = 0.02), with males demonstrating a greater risk than females for both [67]. The other 12 subgroup analyses demonstrated non-significant tests of interaction and/or CI overlap and meta-regression results were not statistically significant
Colon cancer	50, 55, 190	No statistically significant findings
Rectal cancer	50, 55, 190	Corrao et al. reported a significant effect of gender in modifying the linear effect of alcohol, with women presenting higher slopes than men (beta = 0.02824; Z value = 2.361) [64]. The four subgroup analyses examining alcohol and rectal cancer were all uninterpretable
Larynx cancer	12, 13, 55	No statistically significant findings
Lung cancer	11, 13, 41, 50, 346	No statistically significant findings
Urinary tract cancer	332	No statistically significant findings
Kidney cancer	13, 22, 48, 50, 246, 309	No statistically significant findings
Bladder cancer	13, 50, 108, 133, 181, 203, 275	Among 17 subgroup analyses, one showed non-overlap of CIs; for the exposure of any alcohol consumption vs. none, the RR for males was 1.23 (95% CI: 1.13–1.35) and for females was 0.93 (95% CI: 0.82–1.04) [68]. All the other subgroup analyses had non-significant tests of interaction, overlapping CIs, or were uninterpretable
Thyroid cancer	50, 107, 288	No statistically significant findings
Brain cancer	78	The one subgroup analysis found significant heterogeneity between men, women, and men and women subgroups (*p* < 0.001) [69]
Glioma	241	No statistically significant findings
Melanoma	13, 50, 79, 220	No statistically significant findings
Basal cell carcinoma	323	No statistically significant findings
Squamous cell carcinoma	323	No statistically significant findings
Hematologic cancers	50	No statistically significant findings
Leukemia	218	No statistically significant findings
Non-Hodgkin’s lymphoma	13, 208, 263	No statistically significant findings
Multiple myeloma	206, 219	No statistically significant findings
*Alcohol cessation*		
Gastric cancer	115	No statistically significant findings
Multiple myeloma	206	No statistically significant findings

CI, confidence interval; RR, relative risk

### WCRF/AICR recommendation: not smoking, and staying safe in the sun, are also important to reduce your cancer risk

#### Tobacco exposure

Sex/gender findings were identified for tobacco smoking from 46 systematic reviews, in reference to 36 cancer sites. While most systematic reviews did not report significant sex/gender differences, we found that significant sex/gender differences were reported for total cancer, gastric cancer, lung cancer, lung squamous cell carcinoma, lung adenocarcinoma, kidney cancer, melanoma, and acute myeloid leukemia. However, for most associations with significant sex/gender differences, we also identified systematic reviews with no significant sex/gender differences. Findings are described in Table 11. A total of six systematic reviews with sex/gender findings were identified for smokeless tobacco, which were for four associations of interest. Significant sex/gender differences were identified for smokeless tobacco and oral cancer, oropharyngeal cancer, and esophageal cancer, but not pharyngeal cancer. Table 12 describes findings for smokeless tobacco, second-hand smoking, and smoking cessation. For second-hand smoking, we found eight systematic reviews with sex/gender findings, for four associations of interest. No significant sex/gender findings were identified. For smoking cessation, we included results from 21 systematic reviews. These contributed to 18 associations of interest, of which 15 had no significant sex/gender differences. The three cancer sites that did have significant sex/gender differences with regards to smoking cessation were colorectal cancer, lung cancer, and acute myeloid leukemia.

**Table 11:** Summary of sex/gender findings from systematic reviews with quantitative synthesis for tobacco smoking and non-sex-specific cancers

Cancer	Corresponding systematic reviews in Supplemental Table 5	Narrative synthesis of sex/gender findings
Total cancer	113	Inoue et al. reported non-overlap of CIs in the one subgroup analysis (RR men: 1.64 (95% CI: 1.55–1.73); RR women: 1.34 (95% CI: 1.24–1.43)) [70]
Head and neck cancer	130	No statistically significant findings
Upper aerodigestive tract cancer	8	No statistically significant findings
Nasopharyngeal cancer	172, 311	No statistically significant findings
Laryngeal cancer	201	No statistically significant findings
Esophageal cancer	201	No statistically significant findings
Esophageal squamous cell carcinoma	285	No statistically significant findings
Esophageal adenocarcinoma	285	No statistically significant findings
Gastric cancer	132, 159, 196	One of five sex/gender subgroup analyses demonstrated non-overlap of CIs with the RR among men higher than among women [71]. Ladeiras-Lopes et al. reported that summary RR estimates were independently associated with sex (*p* < 0.001), being lower in females relative to males in meta-regression [72]
Gallbladder cancer	174	No statistically significant findings
Liver cancer	150, 201	No statistically significant findings
Pancreatic cancer	114, 173, 182, 201, 361	No statistically significant findings
Colorectal cancer	25, 26, 112, 201, 265	No statistically significant findings
Lung cancer	148, 149, 199, 201, 277, 331	Six RRRs from two systematic reviews reported higher RRs among males relative to females [73, 74]. Lee et al. reported statistically significant differences from subgroup analyses in one systematic review between male, female, and combined groups when comparing ever smoking vs. never or non-current smoking and current smoking vs. never or non-current smoking (both *p* < 0.001) [73], as well as when comparing current vs. never smokers in a different systematic review (*p* < 0.001) (75). One other systematic review reported non-overlapping CIs for men and women, with men at a higher risk [76]. Findings from two other systematic reviews for lung cancer included RRRs crossing the null and overlap of CIs
Lung squamous cell carcinoma	148, 331	One subgroup analysis found a significant difference between males, females, and combined groups (*p* < 0.01) for current smoking vs. never or non-current smoking [73]. However, the same systematic review reported an RRR that crossed the null for ever smoking vs. never or non-current smoking, as did one other systematic review
Lung adenocarcinoma	148, 331	One systematic review reported an RRR of 1.43 (1.14–1.78) for male vs. female, significant differences between strata of males, females, and combined from subgroup analyses (*p* < 0.05), and significant male vs. female findings from meta-regression (*p* < 0.05), all when comparing ever vs. never or non-current smoking [73]. The same review also reported significant subgroup analysis and meta-regression findings when comparing current smoking vs. never or non-current smoking, although one other review reported an RRR that crossed the null
Small cell lung cancer	331	No statistically significant findings
Other lung cancer	331	No statistically significant findings
Urinary tract cancer	333	No statistically significant findings
Kidney cancer	57, 111, 168, 201	Of the five subgroup analyses, one reported a significant difference between men and women (p-value for heterogeneity = 0.02) [77], whereas the others reported overlap of CIs or were uninterpretable
Bladder cancer	57, 101, 201, 273	No statistically significant findings
Thyroid cancer	146	No statistically significant findings
Meningioma	42, 69	No statistically significant findings
Glioma	154, 180, 234	No statistically significant findings
Melanoma	247	The one included subgroup analysis reported non-overlap of CIs for ever vs. never smokers [78]
Basal cell carcinoma	152	No statistically significant findings
Hodgkin’s lymphoma	35, 231	No statistically significant findings
Non-Hodgkin’s lymphoma	36, 231	No statistically significant findings
Diffuse large beta cell lymphoma	231	No statistically significant findings
Chronic lymphocytic leukemia/small lymphocytic lymphoma	231	No statistically significant findings
T-cell non-Hodgkin’s lymphoma	231	No statistically significant findings
Nodular sclerosis	231	No statistically significant findings
Multiple myeloma	205	No statistically significant findings
Acute myeloid leukemia	54, 75	Two of five subgroup analyses, both from one systematic review by Colamesta et al. demonstrated non-overlap of CIs: for current vs. never smokers (RR males: 1.77 (95% CI: 1.30–2.42); females: 1.04 (0.85–1.26)) and for ever vs. never smokers (RR males: 1.78 (95% CI: 1.38–2.30); females: 1.06 (0.90–1.23)); the other three had overlapping CIs [79]
Chronic myeloid leukemia	210	No statistically significant findings
Follicular lymphoma	231	No statistically significant findings

CI, confidence interval; RR, relative risk; RRR, relative risk ratio

**Table 12:** Summary of sex/gender findings from systematic reviews with quantitative synthesis for other tobacco exposures and non-sex-specific cancers

Cancer	Corresponding systematic reviews in Supplemental Table 5	Narrative synthesis of sex/gender findings
*Smokeless tobacco*		
Oral cancer	9, 123, 191, 244, 294	Subgroup analyses from two systematic reviews found significant differences when comparing men/males, women/females, and both groups [80, 81], and a RRR from one systematic review [82] indicated an increased risk among females relative to males (RRR: 1.79 (95% CI: 1.21–2.64)). However, two subgroup analyses from two systematic reviews indicated no significant sex/gender differences, according to a test of interaction and CI overlap, respectively
Pharyngeal cancer	244	No statistically significant findings
Oropharyngeal cancer	147	The one identified systematic review reported a meta-regression where sex (male vs. female vs. combined) was a significant factor (*p* = 0.004) [83]
Esophageal cancer	244	The one systematic review’s subgroup analysis reported significant heterogeneity between men, women, and both genders combined subgroups (p for heterogeneity < 0.001) [80]
*Second-hand smoking*		
Total cancer	124	No statistically significant findings
Colorectal cancer	315	No statistically significant findings
Bladder cancer	272, 274	No statistically significant findings
Lung cancer	66, 158, 201, 291	No statistically significant findings
*Smoking cessation*		
Laryngeal cancer	201	No statistically significant findings
Esophageal cancer	201	No statistically significant findings
Esophageal squamous cell carcinoma	285	No statistically significant findings
Esophageal adenocarcinoma	285	No statistically significant findings
Gastric cancer	132, 159	No statistically significant findings
Gallbladder cancer	174	No statistically significant findings
Liver cancer	150, 201	No statistically significant findings
Colorectal cancer	25, 26, 201, 265	Of the four subgroup analyses, Botteri et al. reported a significant test for interaction, with a greater risk among men than women when comparing former vs. never cigarette smokers (p for heterogeneity = 0.03) [84]. That same review reported no significant sex/gender differences when comparing former vs. never smokers and findings from two subgroup analyses from two other systematic reviews had CI overlap or were uninterpretable
Lung cancer	148, 149, 199, 201, 331	Yu et al. reported three RRRs for males vs. females - former smokers vs. non-smokers, < 10 years since quitting vs. not reported, and ≥ 10 years since quitting vs. not reported – each indicating higher RRs among males [74]. Also, one of four subgroup analyses reported significant differences among males, females, and combined groups (p-value between strata < 0.05) [73]. Two other subgroup analyses reported no sex/gender differences according to a test of interaction and a RRR, and a final subgroup analysis was uninterpretable
Lung squamous cell carcinoma	148	No statistically significant findings
Lung adenocarcinoma	148	No statistically significant findings
Urinary tract cancer	333	No statistically significant findings
Kidney cancer	168, 201	No statistically significant findings
Bladder cancer	101, 201, 273	No statistically significant findings
Hodgkin’s lymphoma	231	No statistically significant findings
Non-Hodgkin’s lymphoma	36, 231	No statistically significant findings
Multiple myeloma	205	No statistically significant findings
Acute myeloid leukemia	54	Of the two subgroup analyses, one demonstrated non-overlap of CIs between males and females (RR males: 1.96 (1.31–2.93); females: 1.08 (0.90–1.30)), when comparing former smokers to never smokers [79]; the other demonstrated CI overlap

CI, confidence interval; RR, relative risk; RRR, relative risk ratio

#### Sun exposure

One systematic review provided three sex/gender subgroup analyses for sun exposure and non-Hodgkin’s lymphoma (Table 13). For the exposures of total sunlight exposure time, working days or school days sunlight exposure, and free days or non-school days sunlight exposure, there were no significant sex/gender findings.

**Table 13:** Summary of sex/gender findings from systematic reviews with quantitative synthesis for sun exposure and non-sex-specific cancers

Cancer	Corresponding systematic reviews in Supplemental Table 5	Narrative synthesis of sex/gender findings
Non-Hodgkin’s lymphoma	125	No statistically significant findings

### Quality assessment

**Fig. 6:**
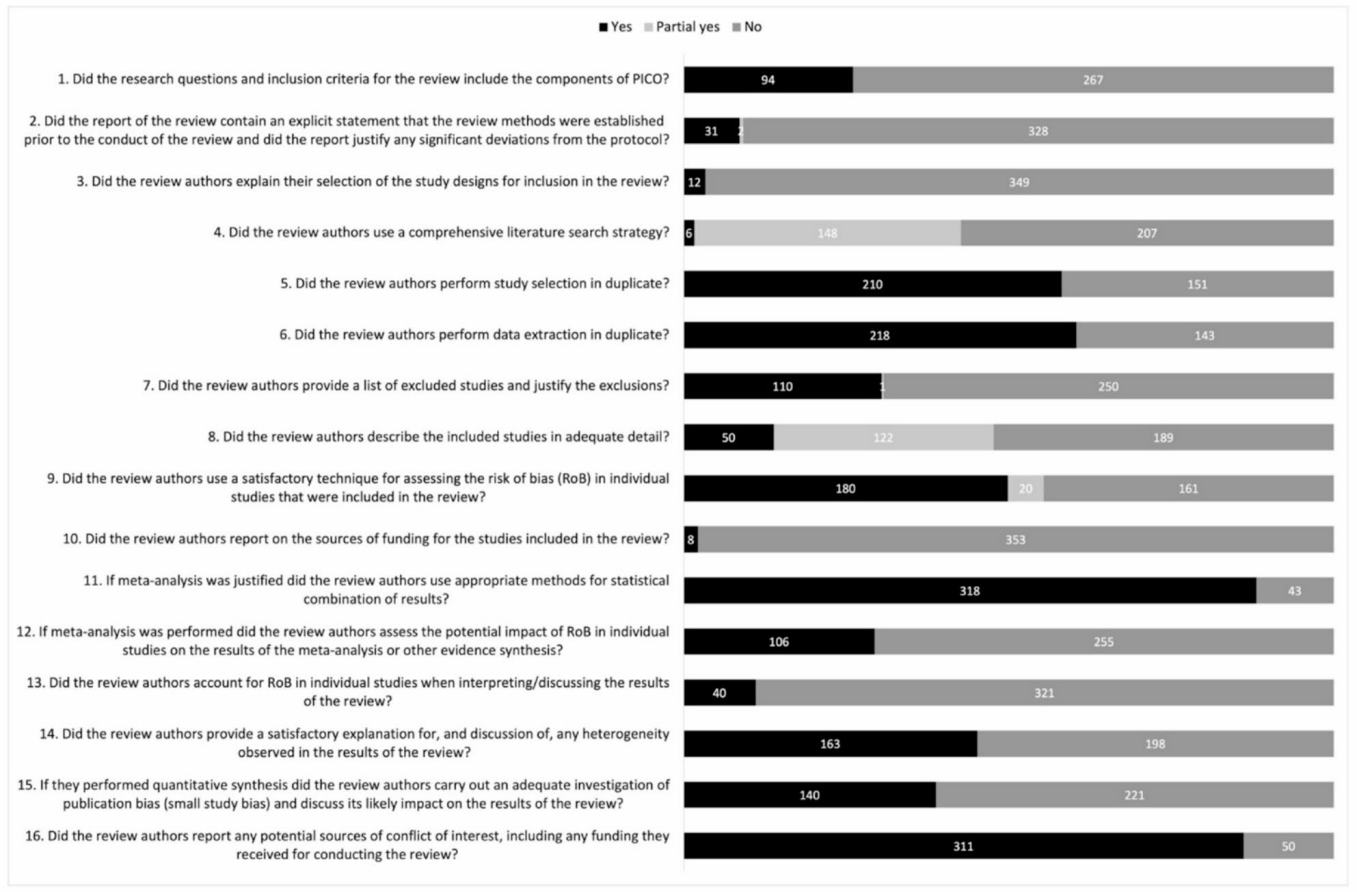
Methodological quality of 361 systematic reviews with sex/gender findings. Quality assessment according to AMSTAR-2 criteria. Abbreviations: PICO, population, intervention, comparison, outcome; RoB, risk of bias

We assessed the methodological quality of the 361 systematic reviews that reported sex/gender findings using the AMSTAR-2 tool. Detailed information on the methodological quality of the reviews is provided in Supplementary Table 6. Based on the seven critical AMSTAR-2 domains, one systematic review was rated “moderate”, four were rated “low”, and 356 were rated “critically low”. The most frequent critical weaknesses identified were not reporting pre-specified methods (checklist item 2; 90.9%), not accounting for risk of bias assessments of primary studies when interpreting findings (checklist item 13; 88.9%), and listing excluded primary studies along with the reason (checklist item 7; 69.3%) (Fig. 6).

## Discussion

### Principal findings

This umbrella review summarized the existing evidence base from systematic reviews with quantitative analysis, on sex/gender associations between behavioural risk factors and non-sex-specific cancers. Numerous reports have described the importance of incorporating sex and gender in health research [85–87]. However, we found that only 361 (51.2%) of the 705 publications that met our inclusion criteria reported quantitative sex/gender associations. Among these 361 systematic reviews, tests for interaction were not statistically significant, CIs overlapped in subgroup analyses, and meta-regression findings were not statistically significant, with few exceptions, when comparing males/men and females/women. While no sex/gender-specific trends were identifiable across behavioural factors and cancers, findings are limited by the critically low quality of most (98.6%) systematic reviews.

Systematic reviews were found reporting sex/gender differences for BMI exposures, with some describing increased risks among females/women (cancers of gallbladder [24–26], lung [24], kidney [24, 32, 41], brain [32], and Hodgkin’s lymphoma [45]) and others among males/men (cancers of liver [24, 27–29], colorectum [24, 31–37], colon [24, 34, 38–40], rectum [24, 32, 38–40], melanoma [40, 42], and leukemia [44]). Systematic reviews were identified with sex/gender differences for other body size exposures, including an increased risk of total cancer among males for bariatric surgery [46], an increased risk of colorectal [47, 48], colon [48], and thyroid cancer [49] among males/men for weight change, and an increased risk among males for colorectal [41] and colon cancer [39] for waist circumference. Sex/gender differences were found for physical activity exposures, including an increased risk among males/men for gastroesophageal [50], gastric [50], and lung cancer [52], as well as sex/gender differences for digestive system [51] and hematologic cancer [53]. One systematic review reported a sex/gender difference for vegetable intake and non-Hodgkin’s lymphoma [54]; another reported sex/gender differences for the DASH diet and colorectal cancer [55]. Sex/gender differences were reported for fish intake and colorectal cancer [56] and salted fish intake and gastric cancer [57], among meat exposures. Regarding coffee, one systematic review reported an increased risk among men [58] and regarding tea, one systematic review reported a protective effect in women [59]. One systematic review was identified reporting a beneficial effect in men for selenium supplementation and total cancer [60], whereas another found an increased risk among studies with majority men for beta-carotene supplements and gastric cancer [61]. Sex/gender differences were also identified for vitamin D supplements and colorectal cancer [62]. For alcohol, a greater risk of oral cavity and pharynx [63], esophageal [65], colorectal [66, 67], and bladder cancer [68] were demonstrated among men, with sex/gender heterogeneity also identified between men, women, and combined sub-groups for brain cancer [69]. For tobacco smoking, sex/gender differences were identified for total cancer [70], gastric cancer [71, 72], lung cancer [73–76], lung squamous cell carcinoma [73], lung adenocarcinoma [73], kidney cancer [77], melanoma [78], and acute myeloid leukemia [79]. Regarding smokeless tobacco, sex/gender differences were reported for oral [80–82], oropharyngeal [83], and esophageal cancer [80]. Increased risks among males/men were identified for the association between smoking cessation and colorectal cancer [84], lung cancer [74], and acute myeloid leukemia [79]. Among the associations for which we did identify systematic reviews with statistically significant sex/gender differences, we typically also identified systematic reviews with no significant sex/gender differences.

The number of systematic reviews with quantitative sex/gender analyses that used the terms “sex” and gender” interchangeably suggests that authors of reviews do not make a distinction between the terms or may be unaware of a difference between them. We used the terms “men and women” or “males and females” in this review in accordance with what the authors of each systematic review used, although it is likely that authors used these terms interchangeably or used “men and women” when examining sex and vice versa. It is also likely that the authors of primary studies included in these systematic reviews are not using the correct sex/gender terminology. Sex/gender analyses in systematic reviews may be based on data pooled from self-report, sex assigned at birth from administrative health data, genotyping data, or other methods. We were unable to verify this, as many systematic reviews did not report which primary studies were included in sex/gender analyses and are effectively a “black box”. As a result, it is unknown whether the few sex/gender differences that we identified in this umbrella review are due to differences in biology or sociocultural effects or whether any sex-specific or gender-specific disparities were masked by pooling multiple dimensions of sex and gender. In turn, interventions for appropriate targeted prevention and healthcare cannot be developed to act upon these disparities [88].

### Strengths and limitations

We have provided a comprehensive summary of the published literature in relation to sex/gender differences in the relationship between behavioural risk factors and incident non-sex-specific cancer. Our choice of risk factors was developed based on guidelines published by the WRCF/AICR [2]. We used systematic and transparent methods, including protocol registration on PROSPERO, an inclusive search strategy of three databases, independent study selection by two authors, and methodological quality assessment using the AMSTAR-2 tool.

Nevertheless, certain limitations of our review should be considered. First, this umbrella review relies on the literature searches conducted in three databases. It is possible that systematic reviews indexed only in other databases were missed, as well as those in grey literature. We restricted to articles published in English, which may have limited the scope. Publication bias is also possible in that systematic reviews and primary studies may have selectively reported statistically significant differences, also known as outcome reporting bias. Second, all included systematic reviews were of moderate, low, or critically low methodological quality on the AMSTAR-2 scale. Previous umbrella reviews have reported that AMSTAR-2 produces a high proportion of critically low ratings (for example, see [89, 90, 91]. Others have suggested that AMSTAR-2 may have poor discriminatory power or that systematic reviews tend to be of low quality [92]. AMSTAR-2 does not include any questions on subgroup analyses or other measures of heterogeneity, which were the focus of our systematic review. Future umbrella reviews may consider alternative assessment tools, such as ROBIS [93]. Third, while we had planned to address overlapping primary studies in this review, we were unable to, as many systematic reviews did not list the primary studies included in their sex/gender analyses. Fourth, subgroup analyses and meta-regression in systematic reviews have inherent limitations. The Cochrane Handbook for Systematic Reviews of Interventions specifies that reliable conclusions may only be drawn from pre-specified analyses and that subgroup analyses and meta-regression conducted after a systematic review has identified heterogeneity should only be used for hypothesis generation and interpreted with caution [94]. As only 33 of 361 systematic reviews with sex/gender results pre-specified or registered a protocol, our findings should be interpreted with caution. Finally, the Cochrane Handbook also recommends that when conducting subgroup analyses, researchers should use a formal statistical test to compare them. Many of the systematic reviews with sex/gender subgroup analyses that we identified did not statistically compare subgroups. Non-overlap of CIs indicates statistical significance when there are only two subgroups, although CIs can overlap a small amount and still be significant [94]. The systematic reviews with subgroup analyses of three groups (e.g., males, females, and combined) without a statistical test that were included in this umbrella review cannot be interpreted.

### Future directions

Although this umbrella review identified a large number of systematic reviews on the association between behavioural factors and non-sex-specific cancers, we still found a need for high-quality further research. Specifically, future systematic reviews could benefit from listing excluded primary studies along with reasons for exclusion, considering risk of bias in the interpretation of findings, and assessing publication bias from primary studies to ensure a comprehensive evidence base, which were all noted as critical domain weaknesses on the AMSTAR-2 checklist among included systematic reviews. Assessment of presence and likely impact of publication bias specifically for sex/gender analyses may also contribute to building a robust evidence base for sex/gender findings.

All included 361 systematic reviews examined sex/gender as a male/female or men/women binary, which raises questions about cisgender individuals as the default population and how transgender, gender-diverse, and non-binary individuals fit within this dichotomous framework. Cancer among transgender individuals has been listed as a research priority [95] and there is a need for systematic reviews and primary studies that examine the association between behavioural factors and cancer in trans (transgender, gender-diverse, non-binary), populations. Barriers that can hinder the participation of trans individuals in research have been documented and include small proportions of the general population identifying as trans which may require labour-intensive and costly practices to recruit a sufficiently large sample size, a lack of gender-diverse data being collected through surveys or captured via administrative health records, and mistrust towards the scientific community which may result in reluctance to answer questions about gender identity [96, 97]. Strategies exist for increasing participation of trans individuals in research, which include recruitment via the trans community (e.g., word of mouth, community and social service organizations, trans-oriented social media, community-based health clinics, Pride festivals), leveraging existing cohort studies with gender-diverse measures, advocating for standardized gender identity data collection in cancer registries, conducting qualitative studies to inform quantitative research priorities, communication of study findings to the community, and trans-friendly research staff [96].

Binary male/female or men/women comparisons fail to account for heterogeneity within sex/gender categories and overlap between categories [98]. While we report on certain statistically significant sex/gender differences in behavioural risk factors and incident cancers, it is unclear whether the categories are sufficiently distinct and homogenous to permit public health and policy developments. Ritz et al. have highlighted that findings of difference between sex/gender categories arise from groupings; averages and differences may not apply to all individuals within a specific sex/gender category [98]. The findings from our umbrella review suggest a need for analyses of sex/gender that do not rely on binaries. Additionally, future research may consider exploring cancer risk from an intersectional perspective, considering not only sex/gender, but also sexuality, race and ethnicity, and other factors demonstrated to influence cancer risk.

Some of the challenges that we identified could be improved by more rigorous methods of querying and operationalizing sex and gender, despite inherent limitations of binary categories and recognition that they are entangled. The Cochrane Handbook for Systematic Reviews of Interventions recommends that authors pre-specify characteristics that will be examined in subgroup analyses or meta-regression. Future systematic reviews should follow this guidance, by pre-specifying subgroup analyses or meta-regression by sex or gender in protocols, which is in alignment with the AMSTAR-2 criteria of establishing review methods prior to the conduct of the review [23, 94]. Systematic reviews conducting sex/gender analyses may want to specify in greater detail what variable they are examining when conducting a subgroup analysis or meta-regression by sex or gender. For instance, a systematic review conducting a meta-regression with the percentage of male participants in primary studies as an explanatory variable could define what they mean by “male” in their analysis and justify the pooling of multiple studies’ male participants for the creation of that variable. At the level of primary studies, operationalization of sex and gender variables may be useful for preventing misuse of sex and gender concepts and avoiding biases. Explicit descriptions of sex and gender frameworks used and sex/gender-related objects of interest, such as hormones or behaviours, may enable more precise understandings of sex/gender-related findings [98].

### Public health and policy implications

Despite substantial evidence on sex/gender differences in behavioural cancer risk factors and in cancer incidence, mortality, and response to treatment [3–5, 10, 11, 99–101], guidelines for cancer prevention remain primarily one-size-fits-all, with few personalized sex/gender recommendations [2, 102–104]. Both the WCRF/AICR guidelines and the 4th edition of the European Code Against Cancer recommend breastfeeding for women, with the latter recommending women limit use of hormone replacement therapy [2, 104]. The Cancer Recommendation for Japanese, developed by the Research Group for the Development and Evaluation of Cancer Prevention Strategies in Japan recommend keeping salt consumption < 8 g/day for men and < 7 g/day for women, and maintaining a BMI of 21–27 for middle-aged to elderly men and 21–25 for middle-aged to elderly women [103]. The American Cancer Society Diet and Physical Activity Guideline recommend consumption of no more than 1 drink/day for women and 2 drinks/day for men [102]. Given the critically low quality evidence of systematic reviews in this umbrella review, it is challenging to identify further behaviour-related cancer prevention guidelines tailored by sex/gender. However, we recommend that developers of cancer prevention guidelines, interventions, and policy include perspectives and expertise in sex and gender, and ensure that output is sex/gender-specific or is applicable to all sexes/genders including trans populations.

### Conclusions

While the literature base on behavioural factors and cancer is well-studied, less research has been conducted on heterogeneity by sex/gender in these associations. This umbrella review synthesized current evidence on these sex/gender differences from 361 systematic reviews and evaluated each review’s methodological quality. For most behavioural exposures and incident non-sex-specific cancers, we found no differences between sex/gender categories, however the evidence base is of critically low quality and inadequate for determining true sex/gender differences.

## Supplementary Information

Supplementary Material 1

Supplementary Material 2

Supplementary Material 3

Supplementary Material 4

Supplementary Material 5

Supplementary Material 6

Supplementary Material 7

Supplementary Material 8

## Data Availability

All data generated or analysed during this study are included in this published article and its supplementary information files.

## Abbreviations

AMSTAR-2: Assessing the Methodological Quality of Systematic Reviews-2
BMI: Body mass index
CI: Confidence interval
DASH: Dietary Approaches to Stop Hypertension
OR: Odds ratio
PICO: Population, intervention, comparison, outcome
PRIOR: Preferred Reporting Items for Overviews of Reviews
RoB: Risk of bias
RR: Relative risk
RRR: Relative risk ratio
WCRF/AICR: World Cancer Research Fund/American Institute for Cancer Research

## References

[1] Wild CP, Weiderpass E, Stewart BW, editors. World Cancer Report: Cancer research for cancer prevention. International Agency for Research on Cancer; 2020. NBK606505. 39432694

[2] Clinton SK, Giovannucci EL, Hursting SD, The World Cancer Research Fund/American Institute for Cancer Research Third Expert Report on Diet. Nutrition, physical Activity, and cancer: impact and future directions. J Nutr. 2020;150(4):663–71. 10.1093/jn/nxz268. 31758189 10.1093/jn/nxz268 PMC7317613

[3] Henley SJ, Thomas CC, Sharapova SR, Momin B, Massetti GM, Winn DM, et al. Vital signs: disparities in Tobacco-Related cancer incidence and Mortality - United States, 2004–2013. MMWR Morb Mortal Wkly Rep. 2016;65(44):1212–8. 10.15585/mmwr.mm6544a3. 27832048 10.15585/mmwr.mm6544a3

[4] Hiza HA, Casavale KO, Guenther PM, Davis CA. Diet quality of Americans differs by age, sex, race/ethnicity, income, and education level. J Acad Nutr Diet. 2013;113(2):297–306. 10.1016/j.jand.2012.08.011. 23168270 10.1016/j.jand.2012.08.011

[5] Poirier AE, Ruan Y, Volesky KD, King WD, O’Sullivan DE, Gogna P, et al. The current and future burden of cancer attributable to modifiable risk factors in canada: summary of results. Prev Med. 2019;122:140–7. 10.1016/j.ypmed.2019.04.007. 31078167 10.1016/j.ypmed.2019.04.007

[6] Canadian Institutes of Health Research. How to integrate sex and gender into research. Government of Canada. 2019. Available from: https://cihr-irsc.gc.ca/e/50836.html

[7] Johnson JL, Greaves L, Repta R. Better science with sex and gender: facilitating the use of a sex and gender-based analysis in health research. Int J Equity Health. 2009;8:14. 10.1186/1475-9276-8-14. 19419579 10.1186/1475-9276-8-14 PMC2689237

[8] Lopes-Ramos CM, Quackenbush J, DeMeo DL. Genome-wide sex and gender differences in cancer. Front Oncol. 2020;10:597788. 10.3389/fonc.2020.597788. 33330090 10.3389/fonc.2020.597788 PMC7719817

[9] Springer KW, Stellman JM, Jordan-Young RM. Beyond a catalogue of differences: a theoretical frame and good practice guidelines for researching sex/gender in human health. Soc Sci Med. 2012;74(11):1817–24. 10.1016/j.socscimed.2011.05.033. 21724313 10.1016/j.socscimed.2011.05.033

[10] Cook MB, Dawsey SM, Freedman ND, Inskip PD, Wichner SM, Quraishi SM, et al. Sex disparities in cancer incidence by period and age. Cancer Epidemiol Biomarkers Prev. 2009;18(4):1174–82. 10.1158/1055-9965.EPI-08-1118. 19293308 10.1158/1055-9965.EPI-08-1118 PMC2793271

[11] Edgren G, Liang L, Adami HO, Chang ET. Enigmatic sex disparities in cancer incidence. Eur J Epidemiol. 2012;27(3):187–96. 10.1007/s10654-011-9647-5. 22212865 10.1007/s10654-011-9647-5

[12] Zheng D, Trynda J, Williams C, Vold JA, Nguyen JH, Harnois DM, et al. Sexual dimorphism in the incidence of human cancers. BMC Cancer. 2019;19(1):684. 10.1186/s12885-019-5902-z. 31299933 10.1186/s12885-019-5902-z PMC6625025

[13] Dong M, Cioffi G, Wang J, Waite KA, Ostrom QT, Kruchko C, et al. Sex differences in cancer incidence and survival: a pan-cancer analysis. Cancer Epidemiol Biomarkers Prev. 2020;29(7):1389–97. 10.1158/1055-9965.EPI-20-0036. 32349967 10.1158/1055-9965.EPI-20-0036

[14] Fidler MM, Gupta S, Soerjomataram I, Ferlay J, Steliarova-Foucher E, Bray F. Cancer incidence and mortality among young adults aged 20–39 years worldwide in 2012: a population-based study. Lancet Oncol. 2017;18(12):1579–89. 10.1016/S1470-2045(17)30677-0. 29111259 10.1016/S1470-2045(17)30677-0

[15] Sung H, Ferlay J, Siegel RL, Laversanne M, Soerjomataram I, Jemal A, et al. Global cancer statistics 2020: GLOBOCAN estimates of incidence and mortality worldwide for 36 cancers in 185 countries. CA Cancer J Clin. 2021;71(3):209–49. 10.3322/caac.21660. 33538338 10.3322/caac.21660

[16] Tosakoon S, Lawrence WR, Shiels MS, Jackson SS. Sex differences in cancer incidence rates by race and ethnicity: results from the surveillance, epidemiology, and end results (SEER) registry (2000–2019). Cancers. 2024;16(5):989. 10.3390/cancers16050989. 38473350 10.3390/cancers16050989 PMC10930733

[17] Selvaraj RC, Cioffi G, Waite KA, Jackson SS, Barnholtz-Sloan JS. A pre-cancer analysis of age and sex differences in cancer incidence and survival in the united States, 2001–2020. Cancers. 2025;17(3):378. 10.3390/cancers17030378. 39941747 10.3390/cancers17030378 PMC11815994

[18] Jackson SS, Marks MA, Katki HA, Cook MB, Hyun N, Freedman ND, et al. Sex disparities in the incidence of 21 cancer types: quantification of the contribution of risk factors. Cancer. 2022;128(19):3531–40. 10.1002/cncr.34390. 35934938 10.1002/cncr.34390 PMC11578066

[19] Khan M, Papier K, Pirie KL, Key TJ, Atkins J, Travis RC. Sex differences in cancer incidence: prospective analyses in the UK biobank. Br J Cancer. 2025;133(2):216–26. 10.1038/s41416-025-03028-y. 40341249 10.1038/s41416-025-03028-y PMC12304391

[20] Aromataris E, Fernandez R, Godfrey CM, Holly C, Khalil H, Tungpunkom P. Summarizing systematic reviews: methodological development, conduct and reporting of an umbrella review approach. Int J Evid Based Healthc. 2015;13(3):132–40. 10.1097/XEB.0000000000000055. 26360830 10.1097/XEB.0000000000000055

[21] Gates M, Gates A, Pieper D, Fernandes RM, Tricco AC, Moher D, et al. Reporting guideline for overviews of reviews of healthcare interventions: development of the PRIOR statement. BMJ. 2022;378:e070849. 10.1136/bmj-2022-070849. 35944924 10.1136/bmj-2022-070849 PMC9361065

[22] Neal RD, Tharmanathan P, France B, Din NU, Cotton S, Fallon-Ferguson J, et al. Is increased time to diagnosis and treatment in symptomatic cancer associated with poorer outcomes? Systematic review. Br J Cancer. 2015;112(Suppl 1):S92–107. 10.1038/bjc.2015.48. 25734382 10.1038/bjc.2015.48 PMC4385982

[23] Shea BJ, Reeves BC, Wells G, Thuku M, Hamel C, Moran J, et al. AMSTAR 2: a critical appraisal tool for systematic reviews that include randomised or non-randomised studies of healthcare interventions, or both. BMJ. 2017;358:j4008. 10.1136/bmj.j4008. 28935701 10.1136/bmj.j4008 PMC5833365

[24] Xue K, Li FF, Chen YW, Zhou YH, He J. Body mass index and the risk of cancer in women compared with men: a meta-analysis of prospective cohort studies. Eur J Cancer Prev. 2017;26(1):94–105. 10.1097/CEJ.0000000000000231. 27662398 10.1097/CEJ.0000000000000231

[25] Larsson SC, Wolk A. Obesity and the risk of gallbladder cancer: a meta-analysis. Br J Cancer. 2007;96(9):1457–61. 10.1038/sj.bjc.6603703. 17375043 10.1038/sj.bjc.6603703 PMC2360167

[26] Liu H, Zhang Y, Ai M, Wang J, Jin B, Teng Z, et al. Body mass index can increase the risk of gallbladder cancer: a meta-analysis of 14 cohort studies. Med Sci Monit Basic Res. 2016;22:146–55. 10.12659/msmbr.901651. 27899789 10.12659/MSMBR.901651 PMC5134363

[27] Chen Y, Wang X, Wang J, Yan Z, Luo J. Excess body weight and the risk of primary liver cancer: an updated meta-analysis of prospective studies. Eur J Cancer. 2012;48(14):2137–45. 10.1016/j.ejca.2012.02.063. 22446023 10.1016/j.ejca.2012.02.063

[28] Larsson SC, Wolk A. Overweight, obesity and risk of liver cancer: a meta-analysis of cohort studies. Br J Cancer. 2007;97(7):1005–8. 10.1038/sj.bjc.6603932. 17700568 10.1038/sj.bjc.6603932 PMC2360408

[29] Yao KF, Ma M, Ding GY, Li ZM, Chen HL, Han B, et al. Meta-analysis reveals gender difference in the association of liver cancer incidence and excess BMI. Oncotarget. 2017;8(42):72959–71. 10.18632/oncotarget.20127. 29069840 10.18632/oncotarget.20127 PMC5641183

[30] Yang C, Lu Y, Xia H, Liu H, Pan D, Yang X, et al. Excess body weight and the risk of liver cancer: systematic review and a meta-analysis of cohort studies. Nutr Cancer. 2020;72(7):1085–97. 10.1080/01635581.2019.1664602. 31544511 10.1080/01635581.2019.1664602

[31] Dai Z, Xu YC, Niu L. Obesity and colorectal cancer risk: a meta-analysis of cohort studies. World J Gastroenterol. 2007;13(31):4199–206. 10.3748/wjg.v13.i31.4199. 17696248 10.3748/wjg.v13.i31.4199 PMC4250618

[32] Fang X, Wei J, He X, Lian J, Han D, An P, et al. Quantitative association between body mass index and the risk of cancer: a global meta-analysis of prospective cohort studies. Int J Cancer. 2018;143(7):1595–603. 10.1002/ijc.31553. 29696630 10.1002/ijc.31553

[33] Huxley RR, Ansary-Moghaddam A, Clifton P, Czernichow S, Parr CL, Woodward M. The impact of dietary and lifestyle risk factors on risk of colorectal cancer: a quantitative overview of the epidemiological evidence. Int J Cancer. 2009;125(1):171–80. 10.1002/ijc.24343. 19350627 10.1002/ijc.24343

[34] Ma Y, Yang Y, Wang F, Zhang P, Shi C, Zou Y, et al. Obesity and risk of colorectal cancer: a systematic review of prospective studies. PLoS ONE. 2013;8(1):e53916. 10.1371/journal.pone.0053916. 23349764 10.1371/journal.pone.0053916 PMC3547959

[35] Moghaddam AA, Woodward M, Huxley R. Obesity and risk of colorectal cancer: a meta-analysis of 31 studies with 70,000 events. Cancer Epidemiol Biomarkers Prev. 2007;16(12):2533–47. 10.1158/1055-9965.EPI-07-0708. 18086756 10.1158/1055-9965.EPI-07-0708

[36] Ning Y, Wang L, Giovannucci EL. A quantitative analysis of body mass index and colorectal cancer: findings from 56 observational studies. Obes Rev. 2010;11(1):19–30. 10.1111/j.1467-789X.2009.00613.x. 19538439 10.1111/j.1467-789X.2009.00613.x

[37] Wang J, Yang DL, Chen ZZ, Gou BF. Associations of body mass index with cancer incidence among populations, genders, and menopausal status: a systematic review and meta-analysis. Cancer Epidemiol. 2016;42:1–8. 10.1016/j.canep.2016.02.010. 26946037 10.1016/j.canep.2016.02.010

[38] Harriss DJ, Atkinson G, George K, Cable NT, Reilly T, Haboubi N, et al. Lifestyle factors and colorectal cancer risk (1): systematic review and meta-analysis of associations with body mass index. Colorectal Dis. 2009;11(6):547–63. 10.1111/j.1463-1318.2009.01766.x. 19207714 10.1111/j.1463-1318.2009.01766.x

[39] Larsson SC, Wolk A. Obesity and colon and rectal cancer risk: a meta-analysis of prospective studies. Am J Clin Nutr. 2007;86(3):556–65. 10.1093/ajcn/86.3.556. 17823417 10.1093/ajcn/86.3.556

[40] Renehan AG, Tyson M, Egger M, Heller RF, Zwahlen M. Body-mass index and incidence of cancer: a systematic review and meta-analysis of prospective observational studies. Lancet. 2008;371(9612):569–78. 10.1016/S0140-6736(08)60269-X. 18280327 10.1016/S0140-6736(08)60269-X

[41] Guh DP, Zhang W, Bansback N, Amarsi Z, Birmingham CL, Anis AH. The incidence of co-morbidities related to obesity and overweight: a systematic review and meta-analysis. BMC Public Health. 2009;9:88. 10.1186/1471-2458-9-88. 19320986 10.1186/1471-2458-9-88 PMC2667420

[42] Sergentanis TN, Antoniadis AG, Gogas HJ, Antonopoulos CN, Adami HO, Ekbom A, et al. Obesity and risk of malignant melanoma: a meta-analysis of cohort and case-control studies. Eur J Cancer. 2013;49(3):642–57. 10.1016/j.ejca.2012.08.028. 23200191 10.1016/j.ejca.2012.08.028

[43] Castillo JJ, Reagan JL, Ingham RR, Furman M, Dalia S, Merhi B, et al. Obesity but not overweight increases the incidence and mortality of leukemia in adults: a meta-analysis of prospective cohort studies. Leuk Res. 2012;36(7):868–75. 10.1016/j.leukres.2011.12.020. 22285508 10.1016/j.leukres.2011.12.020

[44] Larsson SC, Wolk A. Overweight and obesity and incidence of leukemia: a meta-analysis of cohort studies. Int J Cancer. 2008;122(6):1418–21. 10.1002/ijc.23176. 18027857 10.1002/ijc.23176

[45] Abar L, Sobiecki JG, Cariolou M, Nanu N, Vieira AR, Stevens C, et al. Body size and obesity during adulthood, and risk of lympho-haematopoietic cancers: an update of the WCRF-AICR systematic review of published prospective studies. Ann Oncol. 2019;30(4):528–41. 10.1093/annonc/mdz045. 30753270 10.1093/annonc/mdz045

[46] Tee MC, Cao Y, Warnock GL, Hu FB, Chavarro JE. Effect of bariatric surgery on oncologic outcomes: a systematic review and meta-analysis. Surg Endosc. 2013;27(12):4449–56. 10.1007/s00464-013-3127-9. 23949484 10.1007/s00464-013-3127-9 PMC4018832

[47] Chen Q, Wang J, Yang J, Jin Z, Shi W, Qin Y, et al. Association between adult weight gain and colorectal cancer: a dose-response meta-analysis of observational studies. Int J Cancer. 2015;136(12):2880–9. 10.1002/ijc.29331. 25395274 10.1002/ijc.29331

[48] Schlesinger S, Lieb W, Koch M, Fedirko V, Dahm CC, Pischon T, et al. Body weight gain and risk of colorectal cancer: a systematic review and meta-analysis of observational studies. Obes Rev. 2015;16(7):607–19. 10.1111/obr.12286. 25925734 10.1111/obr.12286

[49] Youssef MR, Reisner ASC, Attia AS, Hussein MH, Omar M, LaRussa A, et al. Obesity and the prevention of thyroid cancer: impact of body mass index and weight change on developing thyroid cancer – pooled results of 24 million cohorts. Oral Oncol. 2021;112:105085. 10.1016/j.oraloncology.2020.105085. 33171329 10.1016/j.oraloncology.2020.105085

[50] Behrens G, Jochem C, Keimling M, Ricci C, Schmid D, Leitzmann MF. The association between physical activity and gastroesophageal cancer: systematic review and meta-analysis. Eur J Epidemiol. 2014;29(3):151–70. 10.1007/s10654-014-9895-2. 24705782 10.1007/s10654-014-9895-2

[51] Xie F, You Y, Huang J, Guan C, Chen Z, Fang M, et al. Association between physical activity and digestive-system cancer: an updated systematic review and meta-analysis. J Sport Health Sci. 2021;10(1):4–13. 10.1016/j.jshs.2020.09.009. 33010525 10.1016/j.jshs.2020.09.009 PMC7856558

[52] Buffart LM, Singh AS, van Loon EC, Vermeulen HI, Brug J, Chinapaw MJ. Physical activity and the risk of developing lung cancer among smokers: a meta-analysis. J Sci Med Sport. 2014;17(1):67–71. 10.1016/j.jsams.2013.02.015. 23528254 10.1016/j.jsams.2013.02.015

[53] Jochem C, Leitzmann MF, Keimling M, Schmid D, Behrens G. Physical activity in relation to risk of hematologic cancers: a systematic review and meta-analysis. Cancer Epidemiol Biomarkers Prev. 2014;23(5):833–46. 10.1158/1055-9965.EPI-13-0699. 24633143 10.1158/1055-9965.EPI-13-0699

[54] Chen GC, Lv DB, Pang Z, Liu QF. Fruits and vegetables consumption and risk of non-Hodgkin’s lymphoma: a meta-analysis of observational studies. Int J Cancer. 2013;133(1):190–200. 10.1002/ijc.27992. 23238796 10.1002/ijc.27992

[55] Moazzen S, van der Sloot KWJ, Bock GH, Alizadeh BZ. Systematic review and meta-analysis of diet quality and colorectal cancer risk: is the evidence of sufficient quality to develop recommendations? Crit Rev Food Sci Nutr. 2021;61(16):2773–82. 10.1080/10408398.2020.1786353. 32613845 10.1080/10408398.2020.1786353

[56] Schwingshackl L, Schwedhelm C, Hoffmann G, Knüppel S, Preterre AL, Iqbal K, et al. Food groups and risk of colorectal cancer. Int J Cancer. 2018;142(9):1748–58. 10.1002/ijc.31198. 29210053 10.1002/ijc.31198

[57] Wu B, Yang D, Yang S, Zhang G. Dietary salt intake and gastric cancer risk: a systematic review and meta-analysis. Front Nutr. 2021;8:801228. 10.3389/fnut.2021.801228. 34957192 10.3389/fnut.2021.801228 PMC8692376

[58] Galarraga V, Boffetta P. Coffee drinking and risk of lung cancer: a meta-analysis. Cancer Epidemiol Biomarkers Prev. 2016;25(6):951–7. 10.1158/1055-9965.EPI-15-0727. 27021045 10.1158/1055-9965.EPI-15-0727

[59] Sun CL, Yuan JM, Koh WP, Yu MC. Green tea, black tea and colorectal cancer risk: a meta-analysis of epidemiologic studies. Carcinogenesis. 2006;27(7):1301–9. 10.1093/carcin/bgl024. 16638787 10.1093/carcin/bgl024

[60] Bardia A, Tleyjeh IM, Cerhan JR, Sood AK, Limburg PJ, Erwin PJ et al. Efficacy of antioxidant supplementation in reducing primary cancer incidence and mortality: systematic review and meta-analysis. Mayo Clin Proc. 2008;83(1):23–34. 10.4065/83.1.23 10.4065/83.1.23 18173999

[61] Druesne-Pecollo N, Latino-Martel P, Norat T, Barrandon E, Bertrais S, Galan P, et al. Beta-carotene supplementation and cancer risk: a systematic review and meta-analysis of randomized controlled trials. Int J Cancer. 2010;127(1):172–84. 10.1002/ijc.25008. 19876916 10.1002/ijc.25008

[62] Boughanem H, Canudas S, Hernandez-Alonso P, Becerra-Tomás N, Babio N, Salas-Salvadó J, et al. Vitamin D intake and the risk of colorectal cancer: an updated meta-analysis and systematic review of case-control and prospective cohort studies. Cancers (Basel). 2021;13(11):2814. 10.3390/cancers13112814. 34200111 10.3390/cancers13112814 PMC8201292

[63] Turati F, Garavello W, Tramacere I, Pelucchi C, Galeone C, Bagnardi V, et al. A meta-analysis of alcohol drinking and oral and pharyngeal cancers: results from subgroup analyses. Alcohol Alcohol. 2013;48(1):107–18. 10.1093/alcalc/ags100. 22949102 10.1093/alcalc/ags100

[64] Corrao G, Bagnardi V, Zambon A, Arico S. Exploring the dose-response relationship between alcohol consumption and the risk of several alcohol-related conditions: a meta-analysis. Addiction. 1999;94(10):1551–73. 10.1046/j.1360-0443.1999.9410155111.x. 10790907 10.1046/j.1360-0443.1999.9410155111.x

[65] Yu X, Chen J, Jiang W, Zhang D. Alcohol, alcoholic beverages and risk of esophageal cancer by histological type: a dose-response meta-analysis of observational studies. Alcohol Alcohol. 2020;55(5):457–67. 10.1093/alcalc/agaa047. 32484205 10.1093/alcalc/agaa047

[66] Bagnardi V, Rota M, Botteri E, Tramacere I, Islami F, Fedirko V, et al. Alcohol consumption and site-specific cancer risk: a comprehensive dose-response meta-analysis. Br J Cancer. 2015;112(3):580–93. 10.1038/bjc.2014.579. 25422909 10.1038/bjc.2014.579 PMC4453639

[67] Fedirko V, Tramacere I, Bagnardi V, Rota M, Scotti L, Islami F, et al. Alcohol drinking and colorectal cancer risk: an overall and dose-response meta-analysis of published studies. Ann Oncol. 2011;22(9):1958–72. 10.1093/annonc/mdq653. 21307158 10.1093/annonc/mdq653

[68] Lao Y, Li X, He L, Guan X, Li R, Wang Y, et al. Association between alcohol consumption and risk of bladder cancer: a dose-response meta-analysis of prospective cohort studies. Front Oncol. 2021;11:696676. 10.3389/fonc.2021.696676. 34604033 10.3389/fonc.2021.696676 PMC8479110

[69] Galeone C, Malerba S, Rota M, Bagnardi V, Negri E, Scotti L, et al. A meta-analysis of alcohol consumption and the risk of brain tumours. Ann Oncol. 2013;24(2):514–23. 10.1093/annonc/mds432. 23041590 10.1093/annonc/mds432

[70] Inoue M, Tsuji I, Wakai K, Nagata C, Mizoue T, Tanaka K, et al. Evaluation based on systematic review of epidemiological evidence among Japanese populations: tobacco smoking and total cancer risk. Jpn J Clin Oncol. 2005;35(7):404–11. 10.1093/jjco/hyi114. 15987784 10.1093/jjco/hyi114

[71] Nishino Y, Inoue M, Tsuji I, Wakai K, Nagata C, Mizoue T, et al. Tobacco smoking and gastric cancer risk: an evaluation based on a systematic review of epidemiologic evidence among the Japanese population. Jpn J Clin Oncol. 2006;36(12):800–7. 10.1093/jjco/hyl112. 17210611 10.1093/jjco/hyl112

[72] Ladeiras-Lopes R, Pereira AK, Nogueira A, Pinheiro-Torres T, Pinto I, Santos-Pereira R, et al. Smoking and gastric cancer: systematic review and meta-analysis of cohort studies. Cancer Causes Control. 2008;19(7):689–701. 10.1007/s10552-008-9136-z. 18293090 10.1007/s10552-008-9132-y

[73] Lee PN, Forey BA, Coombs KJ. Systematic review with meta-analysis of the epidemiological evidence in the 1900s relating smoking to lung cancer. BMC Cancer. 2012;12:385. 10.1186/1471-2407-12-385. 22943444 10.1186/1471-2407-12-385 PMC3505152

[74] Yu Y, Liu H, Zheng S, Ding Z, Chen Z, Jin W, et al. Gender susceptibility for cigarette smoking-attributable lung cancer: a systematic review and meta-analysis. Lung Cancer. 2014;85(3):351–60. 10.1016/j.lungcan.2014.07.004. 25064415 10.1016/j.lungcan.2014.07.004

[75] Lee PN, Forey BA, Thornton AJ, Coombs KJ. The relationship of cigarette smoking in Japan to lung cancer, COPD, ischemic heart disease and stroke: A systematic review. F1000Res. 2018;7:204. 10.12688/f1000research.14002.1. 30800285 10.12688/f1000research.14002.1 PMC6367657

[76] Wakai K, Inoue M, Mizoue T, Tanaka K, Tsuji I, Nagata C, et al. Tobacco smoking and lung cancer risk: an evaluation based on a systematic review of epidemiological evidence among the Japanese population. Jpn J Clin Oncol. 2006;36(5):309–24. 10.1093/jjco/hyl025. 16735374 10.1093/jjco/hyl025

[77] Liu X, Peveri G, Bosetti C, Bagnardi V, Specchia C, Gallus S, et al. Dose-response relationships between cigarette smoking and kidney cancer: A systematic review and meta-analysis. Crit Rev Oncol Hematol. 2019;142:86–93. 10.1016/j.critrevonc.2019.07.019. 31387065 10.1016/j.critrevonc.2019.07.019

[78] Song F, Qureshi AA, Gao X, Li T, Han J. Smoking and risk of skin cancer: a prospective analysis and a meta-analysis. Int J Epidemiol. 2012;41(6):1694–705. 10.1093/ije/dys146. 23064412 10.1093/ije/dys146 PMC3535753

[79] Colamesta V, D’Aguanno S, Breccia M, Bruffa S, Cartoni C, La Torre G. Do the smoking intensity and duration, the years since quitting, the methodological quality and the year of publication of the studies affect the results of the meta-analysis on cigarette smoking and acute myeloid leukemia (AML) in adults? Crit Rev Oncol Hematol. 2016;99:376–88. 10.1016/j.critrevonc.2016.01.003. 26830008 10.1016/j.critrevonc.2016.01.003

[80] Sinha DN, Abdulkader RS, Gupta PC. Smokeless tobacco-associated cancers: A systematic review and meta-analysis of Indian studies. Int J Cancer. 2016;138(6):1368–79. 10.1002/ijc.29884. 26443187 10.1002/ijc.29884

[81] Weitkunat R, Sanders E, Lee PN. Meta-analysis of the relation between European and American smokeless tobacco and oral cancer. BMC Public Health. 2007;7:334. 10.1186/1471-2458-7-334. 18005437 10.1186/1471-2458-7-334 PMC2225413

[82] Mu G, Wang J, Liu Z, Zhang H, Zhou S, Xiang Q, et al. Association between smokeless tobacco use and oral cavity cancer risk in women compared with men: a systematic review and meta-analysis. BMC Cancer. 2021;21(1):960. 10.1186/s12885-021-08691-x. 34452595 10.1186/s12885-021-08691-x PMC8394164

[83] Lee PN, Hamling J. Systematic review of the relation between smokeless tobacco and cancer in Europe and North America. BMC Med. 2009;7:36. 10.1186/1741-7015-7-36. 19638245 10.1186/1741-7015-7-36 PMC2744672

[84] Botteri E, Borroni E, Sloan EK, Bagnardi V, Bosetti C, Peveri G, et al. Smoking and colorectal cancer Risk, overall and by molecular subtypes: A Meta-Analysis. Am J Gastroenterol. 2020;115(12):1940–9. 10.14309/ajg.0000000000000803. 32773458 10.14309/ajg.0000000000000803

[85] Bauer GR. Sex and gender multidimensionality in epidemiologic research. Am J Epidemiol. 2023;192(1):122–32. 10.1093/aje/kwac173. 36193856 10.1093/aje/kwac173 PMC9619685

[86] Day S, Mason R, Lagosky S, Rochon PA. Integrating and evaluating sex and gender in health research. Health Res Policy Syst. 2016;14(1):75. 10.1186/s12961-016-0147-7. 27724961 10.1186/s12961-016-0147-7 PMC5057373

[87] Heidari S, Babor TF, De Castro P, Tort S, Curno M. Sex and gender equity in research: rationale for the SAGER guidelines and recommended use. Res Integr Peer Rev. 2016;1:2. 10.1186/s41073-016-0007-6. 29451543 10.1186/s41073-016-0007-6 PMC5793986

[88] Krieger N. Genders, sexes, and health: what are the connections–and why does it matter? Int J Epidemiol. 2003;32(4):652–7. 10.1093/ije/dyg156. 12913047 10.1093/ije/dyg156

[89] Whelan E, Kalliala I, Semertzidou A, Raglan O, Bowden S, Kechagias K, et al. Risk factors for ovarian cancer: an umbrella review of the literature. Cancers (Basel). 2022;14(11). 10.3390/cancers14112708. 10.3390/cancers14112708 PMC9179274 35681688

[90] Yu H, Zhong X, Gao P, Shi J, Wu Z, Guo Z, et al. The potential effect of Metformin on cancer: an umbrella review. Front Endocrinol (Lausanne). 2019;10:617. 10.3389/fendo.2019.00617. 31620081 10.3389/fendo.2019.00617 PMC6760464

[91] Zhao Y, Tang L, Shao J, Chen D, Jiang Y, Tang P, et al. The effectiveness of exercise on the symptoms in breast cancer patients undergoing adjuvant treatment: an umbrella review of systematic reviews and meta-analyses. Front Oncol. 2023;13:1222947. 10.3389/fonc.2023.1222947. 37799468 10.3389/fonc.2023.1222947 PMC10548878

[92] De Santis KK, Pieper D, Lorenz RC, Wegewitz U, Siemens W, Matthias K. User experience of applying AMSTAR 2 to appraise systematic reviews of healthcare interventions: a commentary. BMC Med Res Methodol. 2023;23(1):63. 10.1186/s12874-023-01879-8. 36927334 10.1186/s12874-023-01879-8 PMC10018966

[93] Whiting P, Savović J, Higgins JP, Caldwell DM, Reeves BC, Shea B, et al. ROBIS: A new tool to assess risk of bias in systematic reviews was developed. J Clin Epidemiol. 2016;69:225–34. 10.1016/j.jclinepi.2015.06.005. 26092286 10.1016/j.jclinepi.2015.06.005 PMC4687950

[94] Deeks J, Higgins J, Altman D, McKenzie J, Veroniki A. In: Higgins J, Thomas J, Chandler J, Cumpston M, Li T, Page M, et al. editors. Chapter 10: analysing data and undertaking meta-analyses [last updated November 2024]. Cochrane Handbook for Systematic Reviews of Interventions; 2024. Available from cochrane.org/handbook.

[95] Feldman J, Brown GR, Deutsch MB, Hembree W, Meyer W, Meyer-Bahlburg HF, et al. Priorities for transgender medical and healthcare research. Curr Opin Endocrinol Diabetes Obes. 2016;23(2):180–7. 10.1097/MED.0000000000000231. 26825469 10.1097/MED.0000000000000231 PMC4821501

[96] Owen-Smith AA, Woodyatt C, Sineath RC, Hunkeler EM, Barnwell T, Graham A, et al. Perceptions of barriers to and facilitators of participation in health research among transgender people. Transgend Health. 2016;1(1):187–96. 10.1089/trgh.2016.0023. 28861532 10.1089/trgh.2016.0023 PMC5549538

[97] Zhang Q, Goodman M, Adams N, Corneil T, Hashemi L, Kreukels B, et al. Epidemiological considerations in transgender health: A systematic review with focus on higher quality data. Int J Transgend Health. 2020;21(2):125–37. 10.1080/26895269.2020.1753136. 33015664 10.1080/26895269.2020.1753136 PMC7430478

[98] Ritz SA, Bauer G, Christiansen DM, Duchesne A, Trujillo AK, Maney DL, Operationalization. Measurement, and interpretation of Sex/Gender. In: DuBois LZ, Trujillo AK, McCarthy MM, editors. Sex and gender: toward transforming scientific practice. Springer; 2025. pp. 87–112.

[99] Cook MB, McGlynn KA, Devesa SS, Freedman ND, Anderson WF. Sex disparities in cancer mortality and survival. Cancer Epidemiol Biomarkers Prev. 2011;20(8):1629–37. 10.1158/1055-9965.EPI-11-0246. 21750167 10.1158/1055-9965.EPI-11-0246 PMC3153584

[100] Kim HI, Lim H, Moon A. Sex differences in cancer: Epidemiology, genetics and therapy. Biomol Ther (Seoul). 2018;26(4):335–42. 10.4062/biomolther.2018.103. 29949843 10.4062/biomolther.2018.103 PMC6029678

[101] White AM. Gender differences in the epidemiology of alcohol use and related harms in the united States. Alcohol Res. 2020;40(2):01. 10.35946/arcr.v40.2.01. 33133878 10.35946/arcr.v40.2.01 PMC7590834

[102] Rock CL, Thomson C, Gansler T, Gapstur SM, McCullough ML, Patel AV, et al. American cancer society guideline for diet and physical activity for cancer prevention. CA Cancer J Clin. 2020;70(4):245–71. 10.3322/caac.21591. 32515498 10.3322/caac.21591

[103] Sasazuki S, Inoue M, Shimazu T, Wakai K, Naito M, Nagata C, et al. Evidence-based cancer prevention recommendations for Japanese. Jpn J Clin Oncol. 2018;48(6):576–86. 10.1093/jjco/hyy048. 29659926 10.1093/jjco/hyy048

[104] Schüz J, Espina C, Villain P, Herrero R, Leon ME, Minozzi S, et al. European code against cancer 4th edition: 12 ways to reduce your cancer risk. Cancer Epidemiol. 2015;39(Suppl 1):S1–10. 10.1016/j.canep.2015.05.009. 26164654 10.1016/j.canep.2015.05.009

