# Supplementary figures and images for: Sex/gender differences in the association between behavioural factors and cancers: an umbrella review of systematic reviews with quantitative synthesis
Source: Biol Sex Differ. 2025 Nov 23;16:109. doi: 10.1186/s13293-025-00793-6 (PMC12751745; doi:10.1186/s13293-025-00793-6)

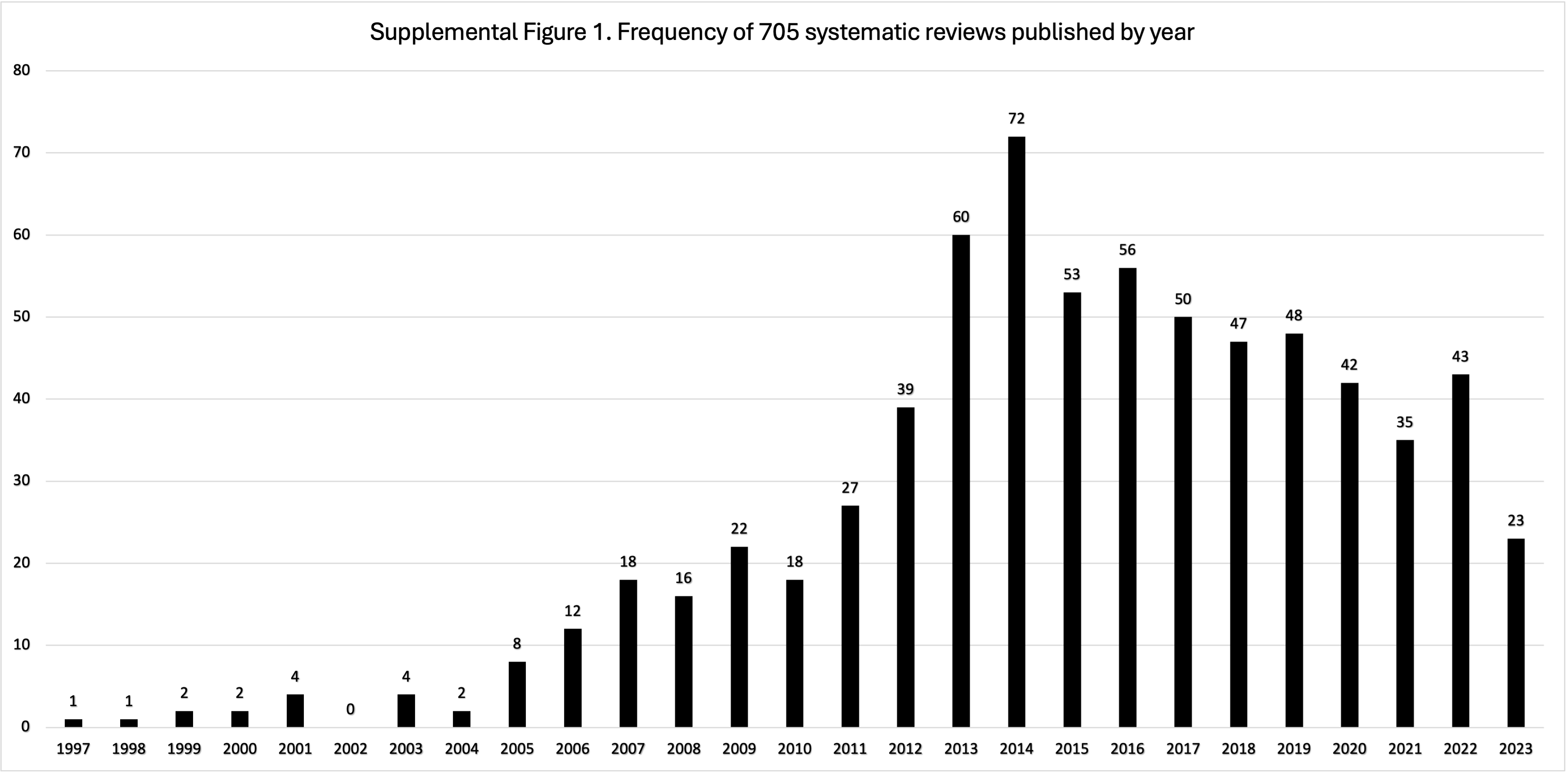

Supplement: Supplementary file 1 — Supplementary Material 1 [file 13293_2025_793_MOESM1_ESM.png]

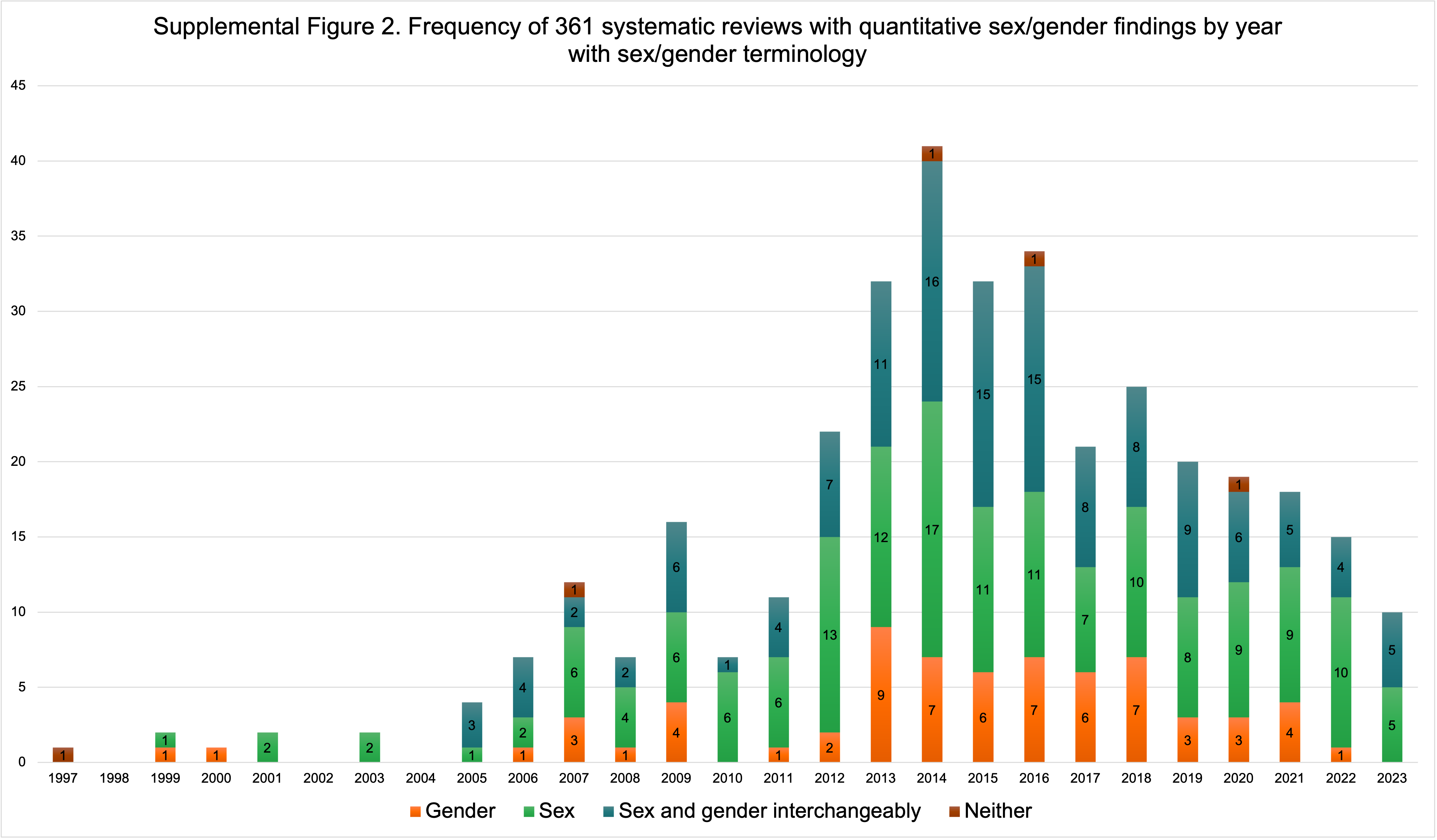

Supplement: Supplementary file 8 — Supplementary Material 8 [file 13293_2025_793_MOESM8_ESM.png]
